# The ADP-binding kinase region of Ire1 directly contributes to its responsiveness to endoplasmic reticulum stress

**DOI:** 10.1038/s41598-021-83890-x

**Published:** 2021-02-24

**Authors:** Quynh Giang Le, Yuki Ishiwata-Kimata, Thi Huong Phuong, Shigeto Fukunaka, Kenji Kohno, Yukio Kimata

**Affiliations:** 1 Division of Bioscience, Graduate School of Science and Technology, Nara Institute of Science and Technology, 8916-5 Takayama, Ikoma, Nara 630-0192 Japan; 2 Institute of Biotechnology, Vietnam Academy of Science and Technology, 18 Hoang Quoc Viet road, Cau Giay, Ha Noi, Vietnam; 3 Graduate School of Life Science, University of Hyogo, 3-2-1 Kouto, Kamigori-cho, Ako-gun, Hyogo 678-1297 Japan

**Keywords:** Cell biology, Molecular biology

## Abstract

Upon endoplasmic-reticulum (ER) stress, the ER-located transmembrane protein, Ire1, is autophosphorylated and acts as an endoribonuclease to trigger the unfolded protein response (UPR). Previous biochemical studies have shown that Ire1 exhibits strong endoribonuclease activity when its cytosolic kinase region captures ADP. Here, we asked how this event contributes to the regulation of Ire1 activity. At the beginning of this study, we obtained a luminal-domain mutant of *Saccharomyces cerevisiae* Ire1, deltaIdeltaIIIdeltaV/Y225H Ire1, which is deduced to be controlled by none of the luminal-side regulatory events. ER-stress responsiveness of deltaIdeltaIIIdeltaV/Y225H Ire1 was largely compromised by a further mutation on the kinase region, D797N/K799N, which allows Ire1 to be activated without capturing ADP. Therefore, in addition to the ER-luminal domain of Ire1, which monitors ER conditions, the kinase region is directly involved in the ER-stress responsiveness of Ire1. We propose that potent ER stress harms cells’ “vividness”, increasing the cytosolic ADP/ATP ratio, and eventually strongly activates Ire1. This mechanism seems to contribute to the suppression of inappropriately potent UPR under weak ER-stress conditions.

## Introduction

The endoplasmic reticulum (ER) is a membrane-bound cellular compartment in which secretory and transmembrane proteins are folded. Furthermore, membrane lipids are biosynthesized mainly on the ER membrane. Dysfunction of the ER, which is mostly caused by, or results in, accumulation of unfolded proteins within the ER, is cumulatively referred to as ER stress and evokes the unfolded protein response (UPR) in eukaryotic cells^1,2^. Ire1 is an ER-located type-I transmembrane protein that acts as an ER-stress sensor to trigger the UPR and is conserved throughout eukaryotic species.

The cytosolic domain of Ire1 has two enzymatic activities as a Ser/Thr protein kinase and an endoribonuclease (supplementary Fig. S1). *HAC1* mRNA is the sole known downstream target of Ire1 in the yeast, *Saccharomyces cerevisiae*. Upon ER stress, Ire1 promotes splicing of *HAC1* mRNA, which is then translated into the transcription factor protein, Hac1, which is responsible for UPR gene regulation, to alleviate the stress conditions^3–5^. According to Kawahara et al. (1997), Hac1 directly binds to the promoter element of the UPR-responsive target genes known as the UPR element (UPRE)^6^.

While the endoribonuclease activity directly contributes to activation of the downstream signaling pathway in the UPR, the role of the kinase region is thought to be regulatory. At least in *S. cerevisiae*, the sole role of Ire1 as a kinase is *trans*-autophosphorylation, in which an Ire1 molecule phosphorylates another self-associated Ire1 molecule^7^. However, it should also be noted that the kinase region of Ire1 has another role that is not associated with phosphotransfer. The nucleotide-binding pocket of the phosphorylated Ire1 molecule captures a nucleotide that acts as a ligand for activation of Ire1 as an endoribonuclease^8,9^. According to Korennykh et al. (2011), ADP works far more effectively than ATP as the activation ligand^10^. Nevertheless, the physiological meaning of this phenomenon remains unclear.

The luminal moiety of Ire1 controls its self-association status and is believed to serve as the ER stress-sensory domain^11^. According to various experimental approaches taken by us and others^12–14^, the luminal domain of *S. cerevisiae* Ire1 has been shown to carry two intrinsically disordered segments, namely Subregions I and V, and one tightly folded segment, which is known as the core luminal domain (cLD; supplementary Fig. S1). Under non-stress conditions, the ER-located molecular chaperone, BiP, is associated with Subregion V, which is juxtamembrane positioned, to suppress the self-association of Ire1^12,15^. The N-terminally positioned segment, Subregion I, also inhibits self-association of Ire1^16^. Upon ER stress, BiP dissociates from Ire1, which then self-associates probably as a dimer^15,17–19^. When dimerized, the cLD forms a groove that is likely to directly capture unfolded proteins accumulated in the ER^14,20^. This event also contributes to the full evocation of the UPR by promoting higher-order oligomerization of Ire1^20,21^.

Nevertheless, as initially suggested by Liu et al. (2000), it is unlikely that the responsiveness of Ire1 to ER stress is only based on its luminal domain^22^. In the present study, we thus asked how Ire1 is regulated dependently by the molecular events on the cytosolic side. We produced *S. cerevisiae* Ire1 mutants that are likely to bypass all of the previously known regulatory events on its luminal side, and demonstrated that they are still controlled in a kinase-region-dependent manner. Our findings illustrate the physiological meaning of the kinase region-dependent regulation of Ire1.

## Results

### The Y225H mutation leads Ire1 to be activated without physical interaction between the cLD and unfolded proteins

**Figure 1:**
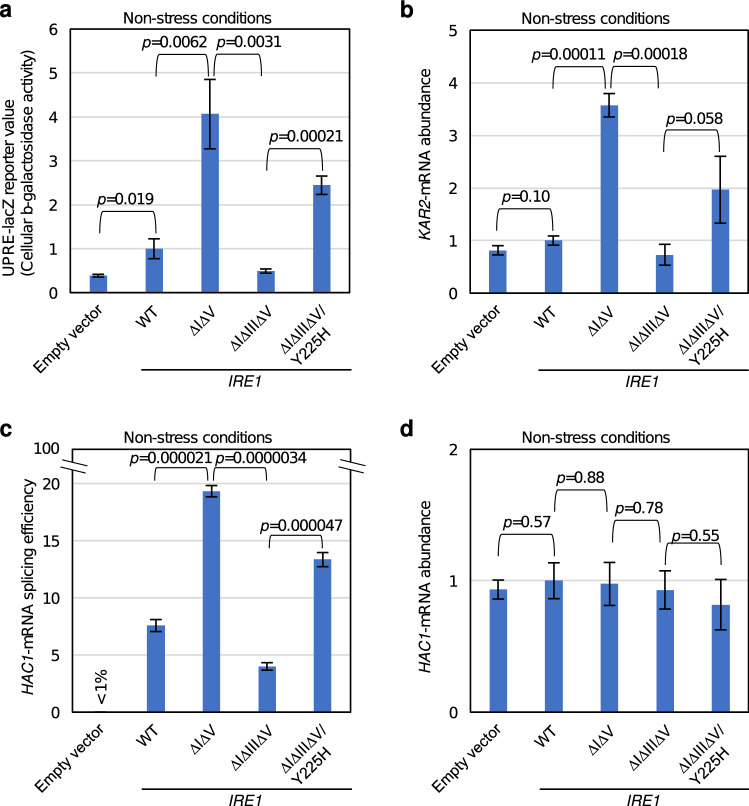
Isolation of the Y225H mutation as an intramolecular suppressor of the low-activity phenotype of ΔIΔIIIΔV Ire1. KMY1015 cells (*ire1Δ*) bearing the UPRE-lacZ reporter plasmid, pCZY1, were transformed with the *IRE1* plasmid, pRS315-IRE1-HA (wild-type: WT), or with that carrying the indicated mutations (or with the empty vector, pRS315), and were grown at 30 °C in SD medium. (**a**) The cultures were subjected to measurement of cellular β-galactosidase activity. (**b**) Total RNA samples were subjected to RT-qPCR analysis to monitor *KAR2*-mRNA abundance. (**c**) Total RNA samples were subjected to RT-PCR to monitor *HAC1*-mRNA splicing. (**d**) Total RNA samples were subjected to RT-qPCR analysis to monitor total *HAC1*-mRNA abundance. In panels (**a**), (**b**), and (**d**), the values are normalized against that of wild-type (WT) *IRE1* cells, which is set at 1.0.

The ΔIΔV mutant of *S. cerevisiae* Ire1, which lacks almost all of subregions I and V, does not undergo repression by these subregions^12,16^, and is highly activated even under non-stress conditions^23^. According to Ref.^20^, even when carrying the ΔIΔV mutation, Ire1 is regulated through direct recognition of unfolded proteins by the cLD^20^. Subregion III (see supplementary Fig. S1 for its position) is a loosely folded loop sticking out from the cLD, the deletion of which impairs the physical interaction between the cLD and unfolded proteins^20,24^. Since the intracellular activity of Ire1 is easily monitored using a UPRE-based reporter, from which β-galactosidase is expressed under the control of the UPRE^25,26^, we reproduced these findings through the UPRE-lacZ reporter assay. As shown in Fig. 1a, the activity of the reporter in unstressed cells was elevated by introduction of the ΔIΔV mutation into Ire1, and was considerably lowered when the ΔIII mutation was further introduced.

The initial step of this study was a screening for Ire1 mutants that intramolecularly suppress this functionless phenotype of the ΔIΔIIIΔV mutation. After random mutagenesis of the *IRE1* gene carrying the ΔIΔIIIΔV triple deletion, as described in the Methods section, we identified an *IRE1* gene thymine673-to-cytosine point mutation, which corresponds to a tyrosine225-to-histidine (Y225H) substitution, (see supplementary Fig. S1 for its position). As shown in Fig. 1a, unstressed cells carrying ΔIΔIIIΔV Ire1 showed high reporter activity when it was further mutated to carry the Y225H mutation. According to the X-ray crystal structure reported by Credle et al. (2005), Y225 is positioned in the β-9 sheet that forms the cLD.

We then asked if the change in the reporter values shown in Fig. 1a actually reflects the induction level of the UPR pathway. As expected, the change in the transcript abundance of *KAR2*, which encodes BiP and is the most prominent UPR-target gene, exhibited a similar pattern to the result from the UPRE-lacZ reporter assay (Fig. 1a,b). Moreover, the same was observed when we checked *HAC1*-mRNA splicing (Fig. 1c). Meanwhile, total *HAC1*-mRNA abundance did not seem to be affected by the Ire1 mutations (Fig. 1d). We think that these observations are reasonable, since the main theme of the UPR signaling pathway is splicing but not transcriptional induction of *HAC1* mRNA.

**Figure 2:**
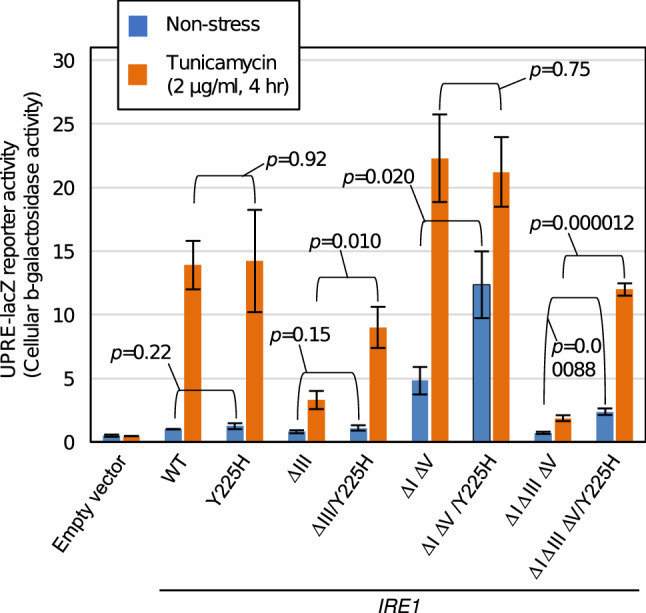
Effect of the Y225H mutation on the UPR inducibility of Ire1 mutants. KMY1015 cells (*ire1Δ*) bearing the UPRE-lacZ reporter plasmid, pCZY1, were transformed with the *IRE1* plasmid, pRS315-IRE1-HA (wild-type: WT), or with that carrying the indicated mutations (or with the empty vector, pRS315), and were grown at 30 °C in SD medium. For ER stress induction, we added tunicamycin (2 µg/mL) into the cultures, which were further cultured for 4 h. The cultures were then subjected to the measurement of cellular β-galactosidase activity, and the resulting values are normalized against that of wild-type (WT) *IRE1* cells, which is set at 1.0.

In the experiment shown in Fig. 2, we introduced the Y225H mutation into wild-type Ire1 and various luminal-domain mutant versions of Ire1, and checked their UPR inducibility using the UPRE-lacZ reporter. The N-glycosylation-inhibiting antibiotic, tunicamycin, is known to impair protein folding in the ER and to induce potent ER stress. Since the reporter values of Y225H-Ire1 cells did not differ from those of wild-type-Ire1 cells under both unstressed and tunicamycin-treated conditions, we think that the Y225H mutation does not largely affect the activity of wild-type Ire1. Meanwhile, the low UPR phenotype of cells carrying ΔIII Ire1 or ΔIΔIIIΔV Ire1 was rescued by introduction of the Y225H mutation.

The UPRE-lacZ reporter assay shown in Fig. 2 also reproduced the aforementioned observation in which ΔIΔV Ire1 exhibits higher activity than wild-type Ire1 under non-stress conditions and is further activated by ER stress^23^. Importantly, the reporter activity was considerably higher in unstressed cells carrying ΔIΔV/Y225H Ire1 in comparison to those carrying ΔIΔV Ire1, although the cellular abundance of ΔIΔV Ire1 was lowered by the Y225H mutation (supplementary Fig. S2). It should also be noted that the reporter activity in cells expressing ΔIΔV/Y225H Ire1 was further elevated by cellular treatment with tunicamycin.

**Figure 3:**
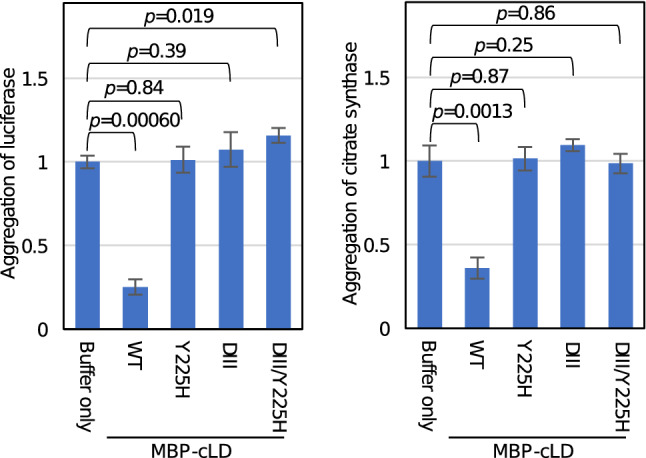
Effect of the Y225H mutation on the physical interaction between the Ire1 luminal domain and unfolded proteins. After denaturation in guanidine HCl solution, luciferase (25 μM) or citrate synthase (50 μM) were 40- (for luciferase) or 100-fold (for citrate synthase) diluted in assay buffer containing MBP-cLD (wild-type: WT) or its mutant versions (1 µM each) or in buffer only, and further incubated at 25 °C for 5 min. The turbidity of the sample mixtures was then measured, normalized against that of the “Buffer only” sample, which is set at 1.0 and presented as “Aggregation”.

According to our previous observation, the ability of the cLD to physically interact with unfolded proteins is observable through in vitro analysis to monitor inhibition of unfolded-protein aggregation by the cLD^20^. Here, we bacterially expressed and purified N-terminal- maltose-binding protein-tagged cLD (MBP-cLD) and its mutant versions (supplementary Fig. S3), and used them in a similar assay to that described in Kimata et al.^20^. In the experiment shown in Fig. 3, two test proteins, citrate synthase and luciferase, were denatured in guanidine HCl-containing solution, and their aggregation was induced through dilution in an assay buffer containing MBP-cLD or its mutants. We then observed that MBP-cLD, but not the ΔIII, Y225H, and ΔIII/Y225H mutants, suppressed the aggregation of denatured citrate synthase and luciferase. This observation suggests that it is unlikely that the Y225H mutation suppresses the low-activity phenotype of the ΔIII mutation by restoring the impaired physical interaction between the cLD and unfolded proteins. Rather, the Y225H mutation seems to compromise the ability of the cLD to capture unfolded proteins.

In order to confirm this idea, we next examined the physical interaction between Ire1 and an ER-located model unfolded protein in *S. cerevisiae* cells. A mutant version of the vacuole protein carboxypeptidase Y (CPY), named CPY*, is known to accumulate in the yeast ER without being properly folded. We previously expressed a green fluorescent protein (GFP)-tagged version of CPY* (CPY*-GFP) in *S. cerevisiae* cells, and found that it is aggregated in the ER and induces the UPR^24^. In the experiment shown in supplementary Fig. S4, cells producing CPY*-GFP were treated with the protein crosslinker dithiobis (succinimidyl propionate) (DSP), lysed, and subjected to anti-GFP immunoprecipitation, which was followed by Western blot analysis for detection of proteins in the immunoprecipitants. The result reproduced our previous finding^24^, in which wild-type Ire1-HA was co-immunoprecipitated when co-expressed with CPY*-GFP. However, such co-immunoprecipitation bands were unobservable when Ire1-HA carried either or both of the ΔIII and Y225H mutations.

Based on these observations, we assume that the Y225H mutation abolishes the requirement of a physical interaction between the cLD and unfolded proteins for activation of Ire1.

### The ΔIΔIIIΔV/Y225H luminal-domain mutant of Ire1 loses its responsiveness to ER stress when the cytosolic-domain kinase-inactive mutation, D797N/K799N, is introduced

The proximal function of Ire1 in the *S. cerevisiae* UPR is the splicing of *HAC1* mRNA, which therefore serves as a finer indicator of Ire1 activation than the UPRE-lacZ reporter. Hereafter, as described previously^24^, we amplified *HAC1* species by subjecting total RNA samples to reverse transcription (RT)-PCR and quantitatively evaluated *HAC1* mRNA-splicing efficiency as described in the Methods section.

**Figure 4:**
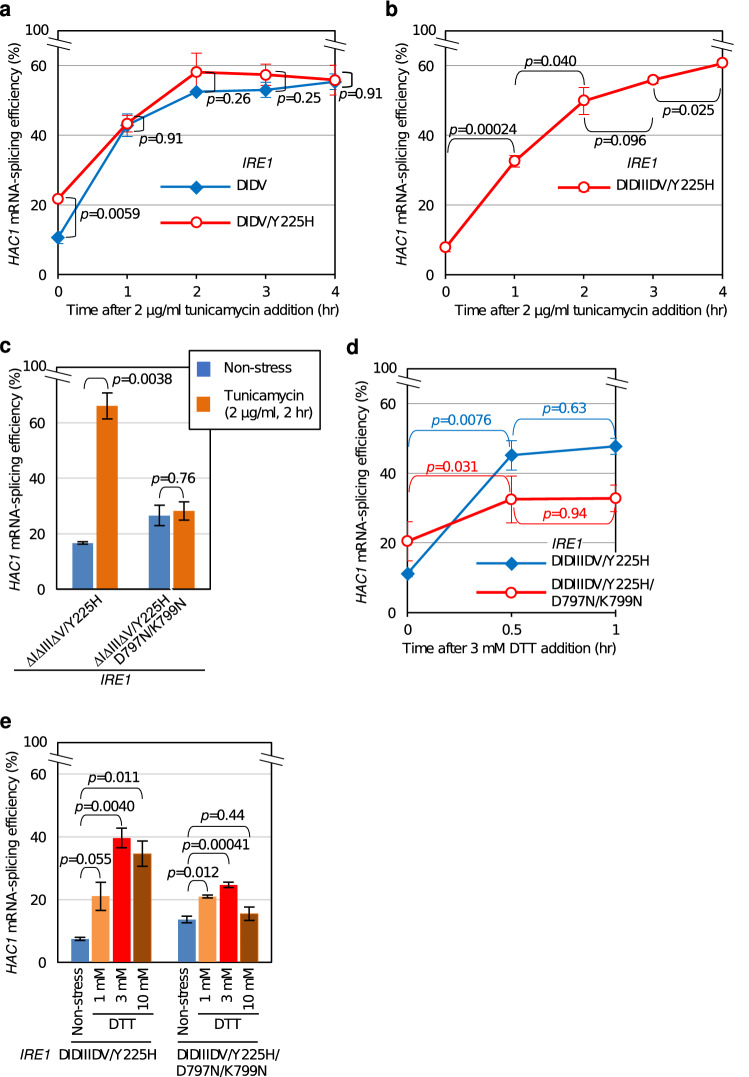
Effect of the D797N/K799N mutation on the ER-stress responsiveness of ΔIΔIIIΔV/Y225H Ire1. After being grown at 30 °C in SD medium, KMY1015 cells (*ire1Δ*) carrying the indicated mutant versions of the *IRE1* plasmid pRS313-IRE1 were ER-stressed and checked for *HAC1*-mRNA splicing efficiency using the RT-PCR technique. (**a** and **b**) Cells were cultured in the presence of 2 µg/mL tunicamycin for the indicated durations. (**c**) Cells were cultured in the presence of 2 µg/mL tunicamycin for 2 h or remained unstressed. (**d**) Cells were cultured in the presence of 3 mM DTT for the indicated durations. (**e**) Cells remained unstressed or were cultured in the presence of 1 or 3 mM DTT for 60 min or 10 mM DTT for 30 min.

The *HAC1* mRNA-splicing profile of cells carrying ΔIΔV Ire1 or ΔIΔV/Y225H Ire1 (Fig. 4a) again demonstrates that the Y225H mutation activates ΔIΔV Ire1 in unstressed cells. Figure 4a also indicates that tunicamycin considerably induces *HAC1*-mRNA splicing in both ΔIΔV-Ire1 cells and ΔIΔV/Y225H-Ire1 cells. A similar result was obtained when we checked the cellular abundance of *KAR2* mRNA (supplementary Fig. S5). As shown in Fig. 4b, ΔIΔV/Y225H Ire1 did not lose responsiveness to tunicamycin even when further mutagenized to carry the ΔIII mutation. Based on our observations presented thus far, we speculate that ΔIΔV/Y225H Ire1 and ΔIΔIIIΔV/Y225H Ire1 are regulated by none of the previously known regulatory events on the luminal side. We then explored the molecular basis underlying the responsiveness of ΔIΔIIIΔV/Y225H Ire1 to ER stress.

The transmembrane domain of Ire1 is known to act as a sensor to monitor the membrane environment and is responsible for UPR induction upon membrane lipid-related aberrations^27^. While, according to Halbleib et al. and Tran et al.^27,28^, this property of Ire1 is abolished upon introduction of the V535R mutation (see supplementary Fig. S1 for its position), the *HAC1*-mRNA splicing of cells expressing ΔIΔIIIΔV/Y225H/V535R Ire1 was sharply induced by cellular treatment with tunicamycin (supplementary Fig. S6). It is therefore unlikely that the ER-stress responsiveness of ΔIΔIIIΔV/Y225H Ire1 is based on the molecular event presented by Halbleib et al.^27^.

Meanwhile, ΔIΔIIIΔV/Y225H Ire1 as well as wild-type Ire1 seem to be able to sense lipid-related aberrations, a prominent example of which is the depletion of inositol from yeast culture. This is because, as shown in supplementary Fig. S7, *HAC1*-mRNA splicing was induced upon inositol depletion in cells carrying ΔIΔIIIΔV/Y225H Ire1 or wild-type Ire1. As expected, the V535R mutation compromised the *HAC1*-mRNA splicing induced by inositol depletion.

According to Rubio et al.^29^, Ire1 is activated without autophosphorylation or binding to ADP when carrying the kinase-inactive mutation D797N/K799N (see supplementary Fig. S1 for its position). As shown in supplementary Fig. S8a, D797N/K799N Ire1 was strictly controlled by ER stress and was upregulated by tunicamycin, although slightly weaker than wild-type Ire1. Meanwhile, in the case of cells carrying ΔIΔIIIΔV/Y225H Ire1, the *HAC1*-splicing efficiency in unstressed cells was increased by introduction of the D797N/K799N mutation (Fig. 4c; Non-stress). Figure 4c also shows that the responsiveness of ΔIΔIIIΔV/Y225H Ire1 to tunicamycin-induced ER stress was almost completely abolished by the D797N/K799N mutation.

The thiol-reducing reagent, dithiothreitol (DTT), is also known to impair protein folding in the ER and cause ER stress when applied into yeast cultures. As well as wild-type-Ire1 cells, cells carrying either ΔIΔIIIΔV/Y225H Ire1 or D797N/K799N Ire1 sharply elevated their *HAC1*-mRNA splicing efficiency in response to DTT exposure (Fig. 4d, e, and supplementary Figs S8b and S9). Meanwhile, when the ΔIΔIIIΔV/Y225H mutation and the D797N/K799N mutation were combined (ΔIΔIIIΔV/Y225H/D797N/K799N-Ire1 cells), the response to DTT exposure was marginal (Fig. 4d, e, and supplementary Fig. S9).

We therefore assume that the responsiveness of ΔIΔIIIΔV/Y225H Ire1 to ER stress is based on the functions of the kinase region of Ire1, which is autophosphorylation and ADP capture.

### The ΔIΔV mutation leads to autophosphorylation of Ire1 even in unstressed cells

**Figure 5:**
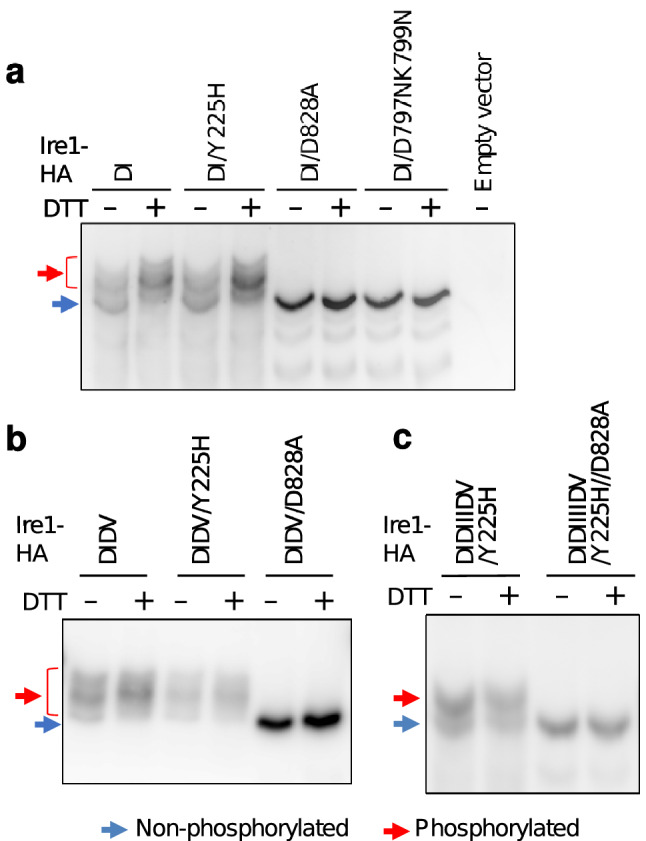
Effect of the Ire1 mutations on its phosphorylation status. After being grown at 30 °C in SD medium, KMY1015 cells (*ire1Δ*) carrying the indicated mutant versions of the Ire1-HA-expression plasmid, pRS315-IRE1-HA, were cultured in the presence of DTT (10 mM, 30 min) or remained unstressed. Lysates of cells (equivalent to OD_600_ = 0.25) were then analyzed by Phos-tag SDS-PAGE and anti-HA Western blotting. The uncropped blots are presented in supplementary Fig. S12.

Phos-tag is an artificial molecule that specifically captures phosphorylated species. On sodium dodecyl sulfate–polyacrylamide gel electrophoresis using Phos-tag-bound polyacrylamide (Phos-tag SDS-PAGE), the migration of phosphorylated proteins is considerably retarded. In order to determine the phosphorylation status of Ire1 tagged with the HA epitope (Ire1-HA), cell lysates were fractionated by Phos-tag SDS-PAGE, which was followed by anti-HA immunoblotting (Fig. 5). In this experiment, cells were ER-stressed with DTT but not with tunicamycin. This is because tunicamycin inhibits N-glycosylation and thus changes the migration of N-glycosylated proteins, such as Ire1, on SDS-PAGE gels independent of their phosphorylation status.

As described in the Methods section, we did not use the metalloprotease inhibitor, ethylenediaminetetraacetic acid (EDTA), when we lysed cells for the Phos-tag SDS-PAGE analysis, because EDTA inhibits the function of Phos-tag to bind to phosphoproteins. Therefore, wild-type Ire1-HA is partially cleaved at Subregion I during cell lysis, causing its complicated band pattern on Phos-tag SDS-PAGE^30^. In the experiment shown in Fig. 5a, we therefore used cells producing ΔI Ire1-HA or its point mutants instead of those producing wild-type Ire1-HA or its point mutants. It should be noted that the stress responsiveness of ΔI Ire1 and ΔI/Y225H Ire1 did not largely differ from that of wild-type Ire1 and Y225H Ire1 (supplementary Fig. S9). As shown in Fig. 5a, migration of ΔI Ire1 on Phos-tag SDS-PAGE was diminished upon stressing of cells with DTT. However, such a shift in migration was not observed when Ire1 carried the kinase-inactive mutation D828A, namely the non-phosphorylated control. When enzymatically dephosphorylated before Phos-tag SDS-PAGE, ΔI Ire1-HA appeared as a sharp singlet band, the migration of which was the same as that of D828A Ire1-HA (supplementary Fig. S10a). No bands were detected from a sample not containing an HA-tagged version of the Ire1 variants (Fig. 5a and supplementary Fig. S10a). We thus assume that, as presented in Ishiwata-Kimata et al.^30^, this method allowed us to monitor the autophosphorylation of Ire1.

Since Y225H/ΔI Ire1-HA exhibited a similar migration shift upon cellular treatment with DTT to that of ΔI Ire1-HA (Fig. 5a), it is unlikely that the Y225H mutation affects the autophosphorylation status of Ire1. Figure 5a also indicates that ΔI Ire1-HA carrying the D797N/K799N mutation remained non-phosphorylated even in the presence of ER stress. This observation is consistent with Rubio et al.^29^, who argued that D797N/K799N Ire1 has the ability to promote *HAC1*-mRNA splicing even though it cannot be autophosphorylated.

Meanwhile, ΔIΔV Ire1-HA or ΔIΔV/Y225H Ire1-HA did not change their migration upon DTT stress (Fig. 5b), although they were upregulated to splice the *HAC1* mRNA under this stress condition (supplementary Fig. S9). In addition to ΔI Ire1-HA, ΔIΔV Ire1-HA appears as a sharp singlet band when carrying the D828A mutation or enzymatically dephosphorylated before Phos-tag SDS-PAGE (Fig. 5b and supplementary Fig. S10b). We therefore assume that the ΔIΔV mutation caused the constitutive autophosphorylation of Ire1. A diffused band pattern of ΔIΔIIIΔV/Y225H Ire1-HA is also likely to indicate its autophosphorylation (supplementary Fig. S10b). Figure 5c shows that the autophosphorylation status of ΔIΔIIIΔV/Y225H Ire1-HA was almost unaffected by ER stress.

Based on their amino-acid sequences, the sizes of ΔI Ire1-HA, ΔIΔV Ire1-HA, and ΔIΔIIIΔV/Y225H Ire1-HA are 120 kDa, 113 kDa, and 111 kDa, respectively. Their migration and point mutants on normal SDS-APGE were almost consistent with the migration of the molecular weight marker (supplementary Fig. S11).

### Activity of ΔIΔIIIΔV/Y225H Ire1 is likely to be linked to levels of cellular ADP

While, according to our observations from the D797N/K799N mutation (Fig. 4c,d,e, and supplementary Fig. S9), ΔIΔIIIΔV/Y225H Ire1 is likely to be regulated through autophosphorylation and/or ADP capture, the autophosphorylation status of ΔIΔIIIΔV/Y225H Ire1 was not regulatory but was constitutive (Fig. 5c). We thus speculate that the ER-stress responsiveness of ΔIΔIIIΔV/Y225H Ire1 is due to its ADP capture. A hypothesis arising from this idea is that ΔIΔIIIΔV/Y225H Ire1 is regulated not by ER accumulation of unfolded proteins per se but by the cytosolic level of ADP.

**Figure 6:**
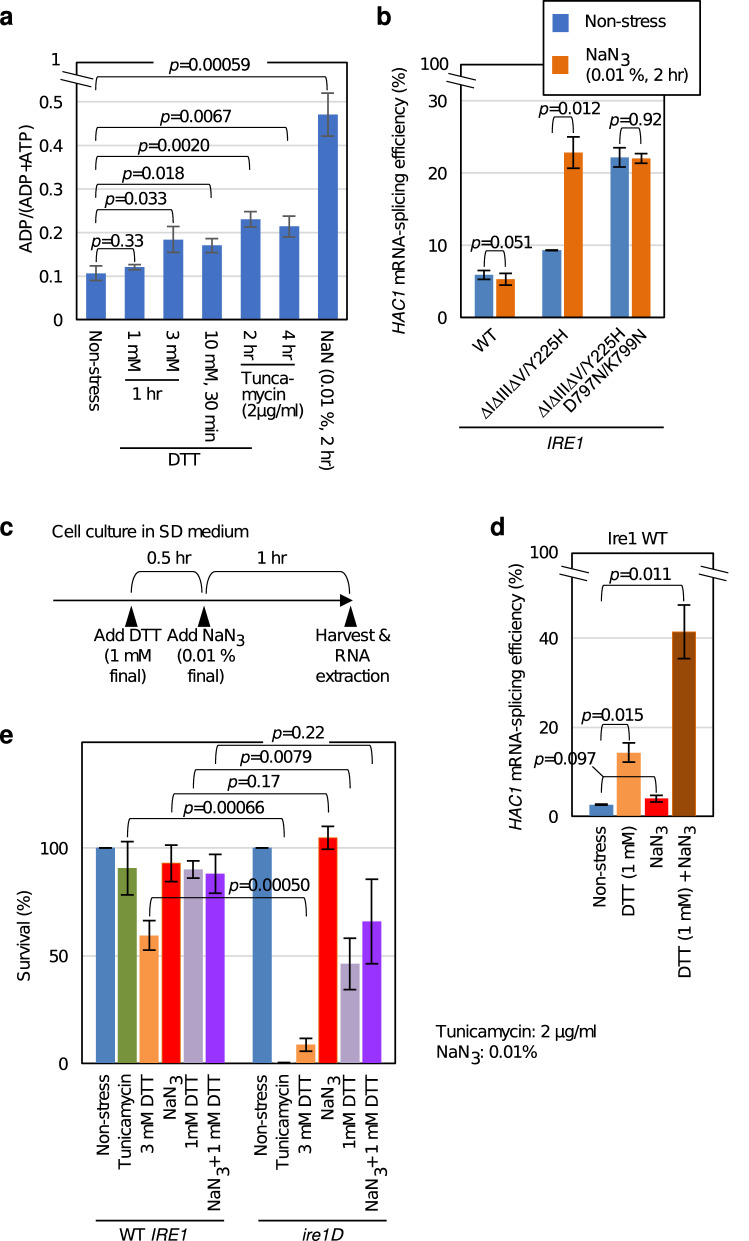
Possible relationship between cellular ADP/ATP ratio and activation of Ire1. (**a**) After being grown at 30 °C in SD medium, KMY1015 cells (*ire1Δ*) carrying the wild-type *IRE1* plasmid, pRS313-IRE1, were further cultured under the indicated stress conditions, and were checked for the cellular ADP/ATP ratio. (**b**) After being grown at 30 °C in SD medium, KMY1015 cells (*ire1Δ*) carrying the *IRE1* plasmid, pRS313-IRE1 (wild-type: WT), or its mutants were further cultured in the presence or absence of 0.01% NaN_3_ for 2 h, and were checked for the *HAC1* mRNA-splicing efficiency using the RT-PCR technique. (**c**) Procedure for dual stressing of cells with dilute DTT and sodium azide. (**d**) KMY1015 cells (*ire1Δ*) carrying the wild-type (WT) *IRE1* plasmid, pRS313-IRE1, were dually stressed as illustrated in panel C, and were checked for the *HAC1* mRNA-splicing efficiency (rightmost column). Alternatively, the cells were treated with either or none of the reagents. (**e**) KMY1015 cells (*ire1Δ*) carrying the wild-type *IRE1* plasmid pRS313-IRE1 (“WT *IRE1*” cells) or the empty vector pRS313 (“*ire1Δ*” cells) were incubated at 30 °C in SD medium in the presence of the indicated chemicals for 3 h. The cultures were then adjusted to OD_600_ = 0.3, serially diluted, and plated on normal SD agar plates, which were incubated for 3 days after which the number of colonies was counted. For the non-stress control, unstressed cultures were similarly treated. The colony-forming unit of each sample is normalized against that of the non-stress control, which is set at 100 and expressed as “Survival (%)”.

In the experiment shown in Fig. 6a, cells were lysed and subjected to a biochemical assay for monitoring of the ADP/ATP ratio. We observed that potent ER-stressing stimuli that strongly activate ΔIΔIIIΔV/Y225H Ire1, namely treatment of cells with 3 or 10 mM DTT or 2 µg/mL tunicamycin, increased the cellular ADP/ATP ratio, although moderately. We did not stress cells with 10 mM DTT for 1 h, because under this condition, cells died quite pronouncedly. Throughout this study, we did not use low concentrations of tunicamycin to induce weak ER stress because, unlike DTT, tunicamycin seems to have a severe threshold concentration to exert any biological effect. On the other hand, we can easily induce weak ER stress by using low concentrations of DTT. Figure 6a also shows that the cellular ADP/ATP ratio was almost unaffected by weak ER stress induced by 1 mM DTT.

We next asked if ΔIΔIIIΔV/Y225H Ire1 is upregulated by a stress stimulus that does not significantly induce ER stress but does highly affect the cellular ADP/ATP ratio. As confirmed in Fig. 6a, sodium azide is known to lead to an increase in the cellular ADP/ATP ratio through inhibition of mitochondrial respiration and other cellular processes^31^. As shown in Fig. 6b, azide treatment induced *HAC1* splicing in cells carrying ΔIΔIIIΔV/Y225H Ire1 but not in cells carrying wild-type Ire1 or ΔIΔIIIΔV/Y225H/D797N/K799N Ire1.

In the experiment shown in Fig. 6c, d, cells carrying wild-type Ire1 were dually stressed by 1 mM DTT, which minimally changed the cellular ADP/ATP ratio (see Fig. 6a), and sodium azide. Strikingly, the dual-stressing stimuli considerably induced *HAC1*-mRNA splicing. We assume that, as described in the Discussion section, this observation can be explained by our argument that Ire1 is dually regulated on the luminal and cytosolic sides. Namely, weak ER stress induced by 1 mM DTT “turns on” the regulatory machinery of Ire1 on the luminal side, whereas sodium azide “turns on” the regulatory event on the cytosolic side by changing the cellular ADP/ATP ratio.

One criticism against this scenario is that azide exposure can be a weak ER-stressing stimulus, which may lead to potent ER stress when combined with 1 mM DTT. In order to address this issue, we performed an experiment as shown in Fig. 6e. Potent ER-stressing stimuli are known to commonly damage *ire1Δ* cells more severely than wild-type *IRE1* + cells^32,33^. This was reproduced in our experiment shown in Fig. 6e, in which *ire1Δ* cells drastically lost their colony-forming ability when strongly ER-stressed by DTT (3 mM, 3 hr) or tunicamycin (2 µg/mL, 3 h). A modest decline in colony-forming ability was also observed when *ire1Δ* cells were stressed by 1 mM DTT for 3 h. Meanwhile, 0.01% sodium azide alone did not seem to affect the colony-forming ability of wild-type *IRE1* cells or *ire1Δ* cells. More importantly, azide exposure did not decrease the colony-forming ability of *ire1Δ* cells even in the presence of 1 mM DTT, suggesting that the combination of azide and 1 mM DTT did not aggravate ER stress. We therefore assume that the potent activation of Ire1 in cells concurrently exposed to azide and 1 mM DTT (Fig. 6d) is not a simple reflection of their ER-stressing status. However, we cannot completely exclude the possibility that azide may induce an imperceptible ER stress, which leads to the activation of a highly sensitive Ire1 variant, ΔIΔIIIΔV/Y225H Ire1.

### Weak ER stress inappropriately activates D797N/K799N Ire1

**Figure 7:**
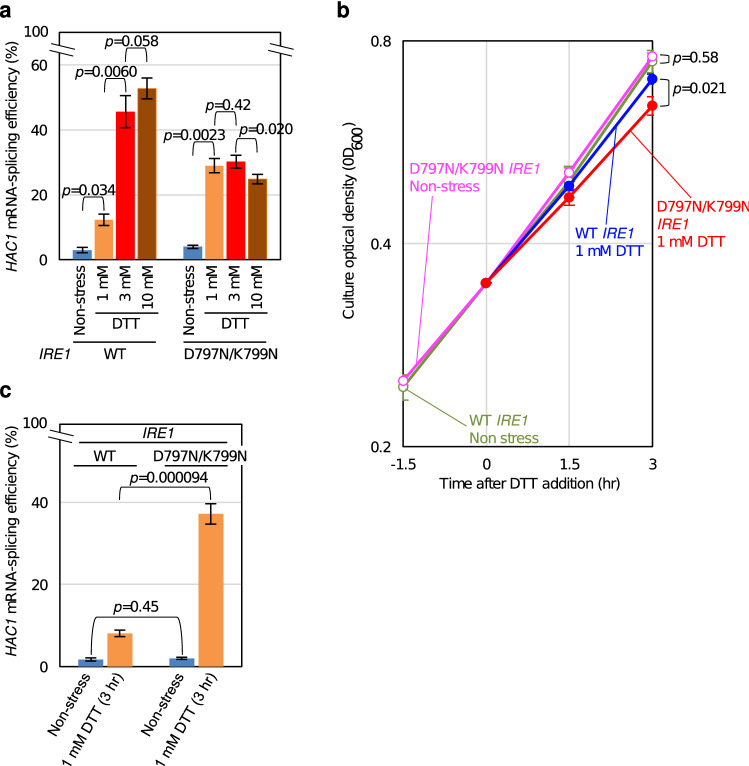
Stress sensitivity of cells carrying either wild-type Ire1 or its D797N/K799N mutant. After being grown at 30 °C in SD medium, KMY1015 cells (*ire1Δ*) carrying the *IRE1* plasmid, pRS313-IRE1 (wild-type: WT), or its D797N/K799N mutant were stressed as follows or remained unstressed. (**a**) Cells were cultured in the absence or presence of DTT (1 or 3 mM for 1 h or 10 mM for 30 min), and were checked for *HAC1* mRNA-splicing efficiency using the RT-PCR technique. (**b**) Optical density (OD_600_) of the cultures was monitored at the indicated time points. For stress imposition, 1 mM DTT was added into cultures at time 0. The values are normalized against that of time 0, which is set at 0.350. (**c**) Cells were cultured in the absence or presence of 1 mM DTT for 3 h, and were checked for *HAC1* mRNA-splicing efficiency using the RT-PCR technique.

In order to explore the physiological meaning of the kinase-domain-dependent regulation of Ire1 more deeply, we finally performed a phenotypic comparison between the wild-type *IRE1* cells and D797N/K799N mutant cells. As shown in Fig. 7a, *HAC1* mRNA-splicing efficiency in cells carrying wild-type Ire1 was increased in a DTT concentration-dependent manner, while D797N/K799N Ire1 cells stressed by 1 mM DTT exhibited high *HAC1* mRNA-splicing efficiency, which was not enhanced by elevating DTT concentration. This observation again argues for the direct involvement of the kinase region in responsiveness of Ire1 to ER-stressing stimuli.

Figure 7b shows that the growth of cells producing D797N/K799N Ire1 was slightly retarded in comparison to that of wild-type Ire1 cells when they were exposed to 1 mM DTT. It should be noted that, even 3-h after stress onset, the D797N/K799N mutation enhanced the efficiency of *HAC1*-mRNA splicing induced by cellular treatment with 1 mM DTT (Fig. 7c). We therefore assume that improperly strong evocation of the UPR leads to growth retardation of D797N/K799N-Ire1 cells under the 1 mM DTT condition.

## Discussion

In general terms, the luminal domain of Ire1 serves as the regulatory unit, which controls RNase activity of the cytosolic domain in response to ER stress. Meanwhile, ΔIΔIIIΔV/Y225H Ire1 still responded to ER stress, although we assume that this mutant is controlled independently of the molecular events on the luminal side (Figs. 1, 2 and 3). Because the stress responsiveness of ΔIΔIIIΔV/Y225H Ire1 was almost completely abolished by the kinase-region mutation, D797N/K799N, we deduce that, in addition to the luminal domain, the kinase region per se contributes to the stress responsiveness of Ire1 (Fig. 4).

One of the roles of the kinase region is the autophosphorylation of Ire1, which is abolished by the D797N/K799N mutation (Fig. 5a; Ref.^29^). The autophosphorylation of Ire1 occurs in response to ER stress^30^ and contributes to the activation of Ire1 as an RNase^7,8^. Nevertheless, we do not think that the stress responsiveness of ΔIΔIIIΔV/Y225H Ire1 is directly linked to its autophosphorylation, because Ire1 variants carrying the ΔIΔV mutation are constitutively autophosphorylated (Fig. 5). Wild-type Ire1 self-associates in an ER-stress dependent manner, while the self-association of ΔIΔV Ire1 is constitutive^13^. Therefore, we deduce that Ire1 is automatically autophosphorylated when it self-associates.

The other role of the kinase region is to capture ADP, which serves as an activation ligand for Ire1^8–10^. Since the kinase domain does not function in phosphotransfer in this case, as initially presented by Papa et al.^34^, non-hydrolyzable nucleotide analogs can function positively for activation of Ire1 or its mutants. Because the D797N/K799N mutation also eliminates the requirement of this process for activation of Ire1^29^, we hypothesize that the stress responsiveness of ΔIΔIIIΔV/Y225H Ire1 is based on its interaction with ADP. In agreement with this idea, ΔIΔIIIΔV/Y225H Ire1 was upregulated by cellular treatment with sodium azide, which inhibits ATP production but does not seem to act as an ER stressor (Fig. 6b). Moreover, our observation in Fig. 6a demonstrates an increase in the cytosolic ADP/ATP ratio alongside potent ER-stressing stimuli, although moderately.

One ATP molecule bound to Ire1 is converted to ADP upon its autophosphorylation. We speculate that this ADP molecule is quickly exchanged with free cytosolic ATP for further autophosphorylation of Ire1, since multiple amino-acid residues are targeted by the Ire1 autophosphorylation^7^. At least in the case of *S. cerevisiae* cells, no report has described phosphorylation of other proteins by Ire1, suggesting that ATP is not hydrolyzed to ADP by the already phosphorylated Ire1 homo-complex. It should be also noted that the in vitro experiments reported by Korennykh et al.^10^ argue that ADP and ATP are captured by the nucleotide-binding pocket of Ire1 with similar affinities, while ADP works far more effectively than ATP as the activation ligand. Thus ATP should serve as a competitive inhibitor of ADP. Based on these insights, we deduce that the kinase region of Ire1 functions as a sensor for the cytosolic ADP/ATP ratio.

Nevertheless, the stress responsiveness of *HAC1* mRNA splicing by ΔIΔIIIΔV/Y225H Ire1 may not be explained solely by the function of the kinase region. As shown in Fig. 4d, e, DTT seemed to upregulate ΔIΔIIIΔV/Y225H/D797N/K799N Ire1, albeit not sharply. Moreover, the *HAC1* mRNA-splicing efficiency of ΔIΔIIIΔV/Y225H Ire1 in azide-treated cells was not as high as that in cells stressed by conventional ER stressors (compare Figs. 4, 5, 6b), although azide treatment elevated the cellular ADP/ATP ratio more drastically than the ER-stressing stimuli (Fig. 6a). Further studies are required to understand the molecular basis underlying these observations.

It should be also noted that, according to our observations presented here (Figs. 4 and 7a, and supplementary Figs S8 and S9) and a previous observation by others^29^, introduction of the D797N/K799N mutation lowers the *HAC1* mRNA-splicing efficiency of fully activated wild-type Ire1 and ΔIΔIIIΔV/Y225H Ire1. This observation is consistent with in vitro biochemical experiments performed by Korennykh et al.^10^, which argues that the RNA-cleavage activity of the Ire1 cytosolic recombinant fragment is compromised by the D797N/K799N mutation.

**Figure 8:**
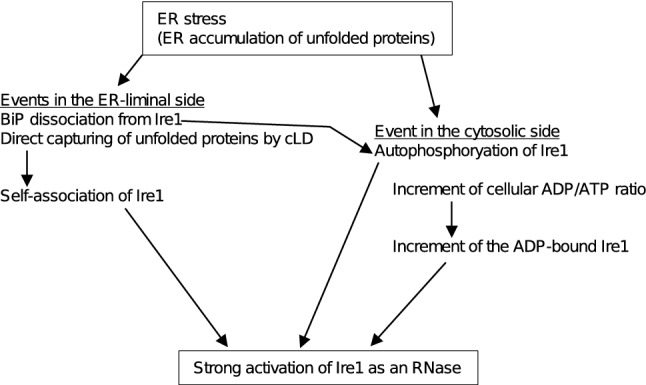
Our model for activation of Ire1 in response to ER stress. On the ER-luminal side, unfolded proteins cause dissociation of BiP from Ire1. Moreover, Ire1 directly captures unfolded proteins. These events cause self-association of Ire1. On the cytosolic side, BiP-unbound and dimerized Ire1 molecules are automatically autophosphorylated. Moreover, potent ER stress increases the cellular ADP/ATP ratio. This also contributes to the activation of Ire1, because ADP serves as an activation ligand of Ire1.

Unlike ΔIΔIIIΔV/Y225H/D797N/K799N Ire1, ΔIΔIIIΔV/Y225H Ire1 and D797N/K799N Ire1 responded sharply to ER stress (Figs. 4 and 7, and supplementary Figs S8 and S9), suggesting that wild-type Ire1 is dually regulated by ER conditions per se and the cytosolic ADP/ATP ratio. Unlike wild-type Ire1, D797N/K799N Ire1, which bypasses regulation by the kinase region, seems to be fully activated even upon weak ER stress, by which the cellular ADP level is not largely affected (Figs. 6a and 7a). Moreover, wild-type Ire1 was strongly activated when cells were dually stressed by 1 mM DTT (weak ER stress) and sodium azide (Fig. 6c, d). We thus propose the following scenario, which is illustrated in Fig. 8, to explain how ER stress leads to activation of Ire1 as an RNase in *S. cerevisiae* cells. Upon ER stress, Ire1 self-associates as it is released from suppression by Subregion V, which serves as the BiP-binding site, and by Subregion^23^. Subsequently, the self-associated Ire1 molecules are automatically autophosphorylated and directly capture ER-accumulated unfolded proteins^20,21^. Moreover, potent ER stress increases the cytosolic ADP/ATP ratio, which is monitored by the kinase region of Ire1, leading to full activation of Ire1.

How does ER stress increase the cytosolic ADP/ATP ratio? Since, as commonly accepted, potent ER stress considerably harms cells, we may not have to assume a sophisticated mechanism. When cells are heavily ER-stressed, they become unhealthy, and various cellular systems may be damaged, which eventually leads to impairment of ATP production. In other words, Ire1 is regulated not only by ER conditions but also by the overall “vividness” of cells, which is reflected by the cytosolic ADP/ATP ratio.

The Ire1-*HAC1* pathway of the UPR is known to control a number of genes in *S. cerevisiae* cells^4,5^. Furthermore, when inappropriately induced, the UPR harms cells^29,35^. Therefore, it seems rational that the UPR remains only weak when ER stress is not potent enough to inhibit ATP biosynthesis. This idea explains the physiological meaning of Ire1 regulation by the cytosolic ADP/ATP ratio. In agreement with this idea, cells carrying D797N/K799N Ire1 exhibited higher *HAC1*-mRNA splicing and retarded growth in comparison to wild-type *IRE1* cells when weakly ER-stressed by 1 mM DTT (Fig. 7).

It should also be noted that *HAC1*-mRNA splicing was poorly induced by treatment of wild-type *IRE1* cells with sodium azide alone (Fig. 6b, d). Moreover, sodium azide did not seem to induce death of wild-type and *ire1Δ* cells (Fig. 6e). Therefore, we do not think that the Ire1-*HAC1* pathway of the UPR is a cellular response that copes with ATP biogenesis defects. An increment of the cytosolic ADP/ATP ratio alone is unlikely to activate wild-type Ire1, which is tightly regulated on its luminal side. On the other hand, our observations shown in Fig. 6 cumulatively indicate that UPR was fully induced by weak ER stress when the cellular ADP/ATP ratio was increased. In other words, the cellular ADP/ATP ratio can affect the sensitivity of Ire1 to ER-stressing stimuli.

In conclusion, the kinase region of Ire1 per se contributes to its responsiveness to ER stress. As aforementioned, Papa et al.^34^ indicated that a kinase-dead and functionless mutant of Ire1 functions in the presence of an ATP analogue, suggesting a role of the Ire1 kinase region that is not directly linked to phosphotransfer. It is currently known that ADP interacts with the ATP-binding pocket of Ire1 and acts as an activation ligand of Ire1^8–10^. Our present study thus explains the physiological meaning of this issue. We propose that the cytosolic ADP/ATP ratio is monitored by the kinase region of Ire1 for fine tuning of the UPR.

## Methods

### Yeast culture

*S. cerevisiae* cells were aerobically and exponentially grown at 30 °C in standard synthetic dextrose (SD) medium containing 2% glucose, 0.66% yeast nitrogen base without amino acids (MP Biomedicals) and appropriate auxotrophic requirements. Alternatively, induction of the *GAL1* promoter was performed as described previously^24^. To stress cells, tunicamycin (2 mg/ml stock in dimethyl sulfoxide), sodium azide (2% stock in water) and DTT (1 M stock in water) were added into the cultures which were then further incubated. The composition of SD medium not containing inositol (SD –ino) was described previously^24^. For the inositol-depletion experiment, cells were exponentially grown in normal SD medium, were washed 6 times with SD (-ino), and were further cultured in SD (-ino).

### Yeast strains and plasmids

In the present study, we employed *S. cerevisiae ire1Δ* strain KMY1015 (*MATα leu2-3,112 ura3-52 his3Δ200 trp1Δ901 lys2-801 ire1::TRP1*; Mori et al., 1996) and its derivatives.

The single-copy *IRE1* plasmid pRS313-IRE1 and the hemagglutinin (HA)-tagged Ire1 (Ire1-HA) plasmid pRS315-IRE1-HA were described previously^12^. Point and partial deletion mutations were introduced into pRS313-IRE1 and pRS315-IRE1-HA as described previously using the overlap PCR and in vivo homologous recombination techniques^12^. See the supplementary Methods for the detailed procedure. The ΔI, ΔIII and ΔV mutations respectively correspond to amino-acid deletions of a.a. 32–91, a.a. 253–272 and a.a. 463–524. For the UPRE-lacZ reporter assay, cells were transformed with plasmid pCZY1^26^. In the experiments shown in Figs. 1, 2 and 5, and supplementary Figs S2, S10, S11 and S12, cells carried pRS315-IRE1-HA or its mutant versions, and thus produced the C-terminal HA-tagged Ire1 or its mutants.

Plasmid pRS313-GAL1pr-CPY*-GFP and a 2µ-based Ire1-HA-expression plasmid pRS426-IRE1-HA are described in Ref.^24^. We introduced *IRE1* mutations into pRS426-IRE1-HA as done for pRS313-IRE1 or pRS315-IRE1-HA. Empty vectors pRS313 and pRS315 are described in Ref.^36^.

### Screening for mutants that intramolecularly suppress the functionless phenotype of ΔIΔIIIΔV Ire1

A randomly mutagenized DNA segment corresponding to the N-terminal half of ΔIΔIIIΔV Ire1, which covers its luminal domain, was obtained by low-fidelity PCR amplification from the ΔIΔIIIΔV *IRE1* gene using the GeneMorph Random Mutagenesis Kit (Stratagene) and the *IRE1*-specfic primer set [P1/P2] (supplementary Table S1). The PCR product DNA (1 µg, 200 ng/µL in TE buffer) and SalI/XbaI-digested pRS315-IRE1-HA (100 ng, 100 ng/µL in TE buffer) were mixed and used for transformation of KMY1015 carrying pCZY1. This procedure generated the circular version of pRS315-IRE1-HA carrying the ΔIΔIIIΔV and random point mutation(s) through in vivo homologous recombination in the transformant cells^12^. For the first screening, transformant colonies were transferred to 5-bromo-4-chloro-3-indolyl β-D-galactopyranoside (X-gal) agar plates^37^, and bluish clones were selected. A second screening was performed via liquid culturing and quantitative cellular β-galactosidase assay of candidate clones.

### Yeast protein analysis

After being harvested through centrifugation at 1,800 Xg for 2 min, 20 OD_600_ cells were broken by agitation (top-speed vortexing at 4 °C for 5 min) with glass beads (425–600 µm) in 200 µl of the lysis buffer (50 mM Tris–Cl (pH7.9), 5 mM EDTA and 1% Triton X-100 supplemented with protease inhibitors, which is described in Ref.^38^). The cell lysates were then clarified by centrifugation at 8,000 Xg for 10 min.

Protein samples were fractionated by the standard Laemmli SDS-PAGE (8% acrylamide) as described in Ref.^38^, and were transferred onto the polyvinylidene difluoride-based Western-bolotting membrane (Hybond-P; GE Healthcare). The semi-dry electrophoretic transfer and subsequent treatment of the blot membranes were performed as indicated by manufacturer’s instruction. The primary antibodies used in this study were 12CA5 mouse monoclonal anti-HA antibody (1 µg/ml), mouse monoclonal anti-GFP antibody (1:1,000 dilution, MBL) and 22C5D8 mouse monoclonal anti-PGK1 antibody (1:2,000 dilution, Abcam). The secondary antibodies used in this study were horseradish peroxidase (HRP)-conjugated goat polyclonal anti-mouse IgG antibody (1:2,000 dilution, Jackson). After application of the enhanced chemiluminescence (ECL) reagent (GE healthcare), we quantitatively detected ECL signal on the blot membranes using the luminescence imager LAS4000 (Fuji Film). Precision Plus Dual Color Standards (Bio-Rad) was employed as the molecular-mass marker for SDS-PAGE.

The Phos-tag SDS-PAGE, which was followed by the anti-HA Western blotting, was performed through the same procedure as done in the standard Laemmli SDS-PAGE, with the following exceptions. The lysis buffer did not contain EDTA, but was supplemented with a phosphatase-inhibitor mixture (Phos-stop, Roche). See supplemental Table S2 for the recipe of the Phos-tag acrylamide gel.

See the supplementary Methods for the procedure employed in the experiment shown in supplementary Fig. S4.

### RNA analyses

We used the hot-phenol method to extract total RNA from cells^38^. Approximately OD_600_ = 5.0 cells were suspended in 1 ml of 50 mM sodium acetate (pH5.3), 10 mMEDTA and 1% EDTA, and were mixed with hot (65 °C) water-saturated phenol. The mixture was then incubated at 65 °C for 30 min with occasional top-speed vortexing, and was fractionated by centrifugation (8,000 Xg, 10 min). The aqueous fraction was subjected to phenol–chloroform extraction twice, and total RNA was precipitated through the standard ethanol-precipitation procedure.

The RNA samples were treated with the AccuRT genomic DNA removal kit (Applied Biological Materials) and were subjected to the reverse-transcription reaction using the ReverTra Ace kit (Toyobo) and the poly(dT)_18_ oligonucletotide primer as described in manufactures’ instructions. The resulting reaction mixture was then employed as a cDNA sample.

In order to monitor *HAC1* splicing, 2 µl of the cDNA samples were subjected to 25 µl-scale PCR using the KAPATaq PCR kit (Nihon Genatics) and 10 pmol each of the *HAC1*-specific primer set (Table S1) in the Thermal cycler PC320 (Astec) that was programmed as follows: 5 min at 96 °C, 25 cycles at 94 °C for 30 s, 54 °C for 30 s and 72 °C for 60 s, and 7 min at 72 °C. The PCR products were fractionated by electrophoresis on 2% agarose gel, the ethidium bromide-stained image of which was used to estimate the *HAC1* mRNA-splicing efficiency, which was calculated using the formula: 100 ×[(band intensity of the spliced form)/{(band intensity of the spliced form) + (band intensity of the unspliced form)}].

For the RT-qPCR analysis, the cDNA samples were subjected to real-time PCR as described in Ref.^38^. The *KAR2*-specific primer set, the *HAC1*-specific primer set and the reference *TAF10*-specific primer set are listed in Table S1.

### In vitro protein aggregation assay

The *Escherichia coli* expression plasmid for MBP-tagged cLD (MBP-cLD), which was previously called MBP-CSSR, is described in our previous publication^20^. MBP-cLD and its mutant versions were expressed in the *E. coli* strain BL21 codon plus (DE3)–RIL (Strategene) and purified as described previously^20^.

In vitro protein aggregation assays were also performed basically as described previously^20^. In brief, luciferase (25 μM final concentration) and citrate synthase (50 μM final concentration) were incubated in the guanidine HCl-denaturing solution (guanidine HCl (6 M for luciferase or 4 M for citrate synthase), 20 mM Hepes, 50 mM KCl, and 2 mM MgCl_2_) for 30 min at room temperature. The denaturing mixture was then diluted with the assay buffer (20 mM Hepes, pH 7.2, 50 mM KCl, and 2 mM MgCl_2_; 40-fold dilution for luciferase and 100-fold dilution for citrate synthase) containing 1 µM MBP-cLD or its mutants. The resulting mixtures were then incubated at 25 °C for 5 min, and protein aggregation was monitored by measuring turbidity (absorbance at 320 nm on a Shimadzu UV-1800 spectrophotometer).

### Other techniques

Culture optical density (OD_600_) was measured using the SmartSpec 3000 spectrophotometer (BioRad).

In order to estimate the cellular ADP/ATP ratio, cellular metabolism was quenched by mixing 100 µl of culture with 200 µl of -40 °C methanol. Cells were lysed by adding 200 µl of acetonitrile into the mixture, which was then agitated with 30 s top-speed vortexing. The resulting cell lysates were dried in a speed vac evaporator and were subjected to the bioluminescent analysis using the EnzyLight ADP/ATP Ratio Assay Kit (BioAssay Systems).

See the supplementary Methods for the procedure of UPRE-lacZ reporter assay. The procedure for the cell survival assay is also described in the supplementary Methods.

### Statistics

For the RNA analyses, the cell survival assay, the growth curve measurement and the UPR-lacZ reporter assays, triplicate biological replicates (three independent transformants with pRS313-IRE1, pRS315-IRE1-HA, their variants or the empty vectors) were assayed per one condition, and results are presented as the averages and standard deviations.

In order to obtain *p* values, we performed two-tail unpaired t-Test (for Figs. 1, 2, 3, 4a, 6e, 7b,c, and supplementary Figs S2, S5 and S7) and paired t-Test (for Figs. 4b–e, 6a,b,d and 7a, and supplementary Figs S6, S8 and S9) using Microsoft Excel.

## Supplementary Information

Supplementary Information
