# Supplementary material for: The ADP-binding kinase region of Ire1 directly contributes to its responsiveness to endoplasmic reticulum stress
Source: Sci Rep. 2021 Feb 24;11:4506. doi: 10.1038/s41598-021-83890-x (PMC7904763; doi:10.1038/s41598-021-83890-x)
Supplement: Supplementary file 1 — Supplementary Information [file 41598_2021_83890_MOESM1_ESM.pdf]

Supplementary information

Title

The ADP-binding kinase region of Ire1 directly contributes to its responsiveness to endoplasmic reticulum stress

Authors

Giang Quynh Le[1,2], Yuki Ishiwata-Kimata[1], Phuong Thi Huong[1], Shigeto Fukunaka[1], Kenji Kohno[3] and Yukio Kimata[1]

[1]Division of Bioscience, Graduate School of Science and Technology, Nara Institute of Science and Technology, 8916-5 Takayama, Ikoma, Nara 630-0192, Japan

[2]Institute of Biotechnology, Vietnam Academy of Science and Technology, 18 Hoang Quoc Viet road, Cau Giay, Ha Noi, Viet Nam

[3]Graduate School of Life Science, University of Hyogo, 3-2-1 Kouto, Kamigori-cho, Ako-gun, Hyogo 678-1297, Japan

Corresponding author
Yukio Kimata
Division of Bioscience, Graduate School of Science and Technology, Nara Institute of Science and Technology, 8916-5 Takayama, Ikoma, Nara 630-0192, Japan
E-mail: kimata@bs.naist.jp

<u>List of supplemental materials</u>

Supplementary methods

Table S1 List of oligonucleotides used in this study

Figure S1 Structure of *S. cerevisiae* Ire1

Figure S2 Effect of the Y225H mutation on cellular abundance of ΔIΔV Ire1

Figure S3 MBP-cLD and its mutants used in Fig. 3.

Figure S4 *In vivo* interaction between Ire1 and a model ER-located unfolded protein is abolished by the ΔIII or the Y225H mutation

Figure S5 Induction of the *KAR2* gene upon tunicamycin exposure in cells carrying ΔIΔIIIΔV or ΔIΔIIIΔV/Y225H Ire1

Figure S6 Effect of the V535R mutation on the *HAC1* mRNA-splicing efficiency

Figure S7 Induction of the *HAC1*-mRNA splicing upon inositol depletion in cells carrying wild-type or ΔIΔIIIΔV/Y225H Ire1

Figure S8 Responsiveness of D797N/K799N Ire1 to potent ER-stress stimuli

Figure S9 Responsiveness of Ire1 mutants to 10 mM DTT

Figure S10 λ-protein phosphatase treatment of the Phos-tag SDS-PAGE samples

Figure S11 Detection of the Ire1-HA mutants through normal SDS-PAGE and anti-HA Western blotting

Figure S12 Uncropped immunoblot images shown in Fig. 5

Supplementary Methods

Introduction of mutations into the IRE1 gene: The D797N/K799N mutant versions of pRS313-IRE1 and pRS315-IRE1-HA were generously provided from Dr. Tran MD (Vietnamese Academy of Science and Technology, Vietnam). In order to introduce point and partial deletion mutations into these plasmids, we used the overlap PCR and *in vivo* homologous recombination techniques through the following procedure. At first, partial *IRE1* DNA fragments were PCR-amplified from pRS313-IRE1 using a primer set [P1/a reverse mutagenic primer] and a primer set [a forward mutagenic primer /P2] (supplementary Table S1). For fusion of the resulting two PCR products, they were mixed and were used as the template for the second PCR in which the primer set [P1/P2] was employed. For the PCR, we used the Pyrobest DNA polymerase (Takara) and the Thermal cycler PC320 (Astec) that was programmed as follows: 25 cycles at 98 °C for 15 sec, 55 °C for 30 sec and 72 °C for 120 sec. Products of the second PCR (1 μg, 200 ng/μL in TE buffer) and SalI/XbaI-digested pRS313-IRE1 or pRS315-IRE1-HA (100 ng, 100 ng/μL in TE buffer) were mixed and used for transformation of KMY1015, the procedure of which is described in Ref. 37.

UPRE-lacZ reporter assay: Cells carrying pCZY1were harvested from 1 ml culture, were suspended in 800 μl of the β-galactosidase reaction buffer (100 mM sodium phosphate buffer, pH 7.0, 10 mM KCl, 1mM $MgSO_4$ and 40 mM 2-mercaptoethanol), and were lysed by adding 20 μl of 0.1% SDS and 50 μl of chloroform into the suspension, which were then top-speed vortexed for 30 sec. The suspension was mixed with 200 μl of 4 mg/ml 2-nitrophenyl-β-D-galactopyranoside (ONPG, the β-galactosidase substrate) solution. The β-galactosidase reaction (conversion of ONPG to 2-nitropheol) was performed at 28 °C, and was halted by adding 500 μl of 1M Na_2CO_3 into the reaction mixture. After being clarified by centrifugation at 8,000 Xg for 5 min, the reaction mixture was checked for optical absorbance at 420 nm (A_{420}) for monitoring concentration of the reaction product 2-nitropheol. The cellular relative β-galactosidase activity was calculated using the formula: (A_{420} after the reaction)/{(OD_{600} of the culture) X (reaction time)}.

Cell survival assay: After stress imposition, cultures were suspended in normal SD medium, and the optical density of the cell suspensions were adjusted to be OD_{600} = 0.50. We also prepared suspensions of unstressed cells for the non-stress control through a similar procedure. The cell suspensions were then serially diluted, and 100 μl of them were plated onto standard 9-mm agar plates (solidified normal SD medium). The agar plates were then incubated at 30 °C for 3 days, and numbers of yeast colonies were counted.

The “Survival (%)” was calculated using the formula: 100X[(number of colonies from stressed sample)X(dilution rate of non-stress control)/{(number of colonies from non-stress

control)X(dilution rate of stressed sample)}.

***In vivo* protein crosslinking and anti-GFP immunoprecipitation:** For the experiment shown in Fig. S4, 25 OD_{600} cells were incubated in 800 µl of PBS containing 2 mM DSP at 25 °C for 1 hr. The cross-linking reaction was then quenched by addition of Tris-HCl (pH 7.5) to 100 mM into the cell suspension, and cells were lysed as described in the main text. For anti-GFP immunoprecipitation, cell lysates were mixed with 800 µl of the immunoprecipitation buffer (50mM Tris-Cl (pH7.9), 5 mM EDTA 150 mM NaCl, 1% Triton X-100) containing 1% skim milk and 4 µl of rabbit polyclonal anti-GFP antibody (MBL). After being incubated at 4 °C for 1hr, this mixture was rotated with 20 µl of the protein-A sepharose (CL-4B) beads (GE Healthcare), which was then washed 5-times with the immunoprecipitation buffer and was boiled with 20 µl of the standard Laemmli SDS-DTT polyacrylamide gel-electrophoresis (PAGE) sampling buffer for 1 min [38].

Table S1 List of oligonucleotides used in this study

IRE1 mutation primers	
P1 (Forward)	CCATTATCACTTTTCTCCATATCA
P2 (Reverse)	CCTTGAAAACTTCCCTGAAAAACT
ΔI mutation (Forward)	TGCTCAATCCCATTGTCGTCTCGCCGTGCTAACAAAAAAGGACGTA
ΔI mutation (Reverse)	TACGTCCTTTTTTGTTAGCACGGCGAGACGACAATGGGATTGAGCA
ΔIII mutation (Forward)	CAGCGTTCGGACCTGGTTCAAAAGAATGTGAAAATATGATTGTAATAGGC
ΔIII mutation (Reverse)	GCCTATTACAATCATATTTTCACATTCTTTTGAACCAGGTCCGAACGCTG
ΔV mutation (Forward)	CACCTTTATGAAAACTATGAAAAACAAAATTCTTTGCTACTGAAGTTTG
ΔV mutation (Reverse)	CAAACTTCAGTAGCAAAGAATTTTGTTTTTCATAGTTTTCATAAAGGTG
Y225H mutation (Forward)	AATTGTTGAAGATGAAAAGGTCCACACTGGATCGATGAGAACTATAA
Y225H mutation (Reverse)	TTATAGTTCTCATCGATCCAGTGTGGACCTTTTCATCTTCAACAATT
V535R mutation (Forward)	TGAAGTTTGGAAGTCTAAGATATCGAATTATAGAG
V535R mutation (Reverse)	CTCTATAATTCGATATCTTAGACTTCCAAACTTCA
RT-PCR and RT-qPCR primers	
Poly(dT)$_{18}$	TTTTTTTTTTTTTTTTTT
HAC1 specific (Forward) [for monitoring splicing]	TACAGGGATTTCCAGAGCACG (Exon 1)
*HAC1*specific (Reverse) [for monitoring splicing]	TGAAGTGATGAAGAAATCATTCAATTC (Exon 2)
KAR2 specific (Forward)	TCTGAAGGTGTCTGCCACAG
KAR2 specific (Reverse)	TTAGTGATGGTGATAGATTCGGATT
HAC1 specific (Forward) [for qPCR]	GCGTCGGACCAAGAGACTT （Exon 1）
*HAC1*specific (Reverse) [for qPCR]	TCGTCGACTCTGGTACATTTTC （Exon 1）
TAF10 specific (Forward)	ATATTCCAGGATCAGGTCTTCCGTAGC
TAF10 specific (Reverse)	GTAGTCTTCTCATTCTGTTGATGTTGTTGTTG

Table S2 Recipe the Phos-tag acrylamide gel

Resolve gel	
30% Acrylamide/Bis solution (Bio Rad)	1.5 ml
1.5 M Tris-Cl (pH8.8)	2.25 ml
10% SDS	90 µl
Water	5.1 ml
Phos-tag acrylamide ALL-107 (Fujifilm)	45 µl
10 mM $MnCl_2$	45 µl
10% Ammonium peroxodisulpahate (APS)	45 µl
Tetramethylethylenediamine	5.7 µl
Stacking gel	
30% Acrylamide/Bis solution (Bio Rad)	0.4 ml
0.5 M Tris-Cl (pH6.8)	0.75 ml
10% SDS	30 µl
Water	1.8 ml
10% Ammonium peroxodisulpahate (APS)	27 µl
Tetramethylethylenediamine	2.9 µl

Sampling buffer (containing 1% DTT) and running buffer are the same as those for standard SDS-PAGE.

Figure S1

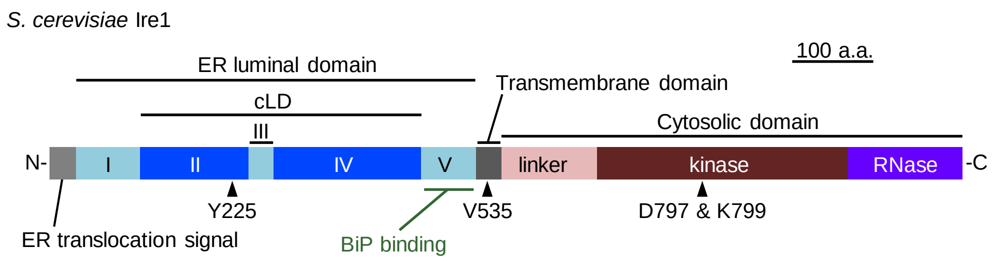

Figure S1 Structure of *S. cerevisiae* Ire1

Figure S2

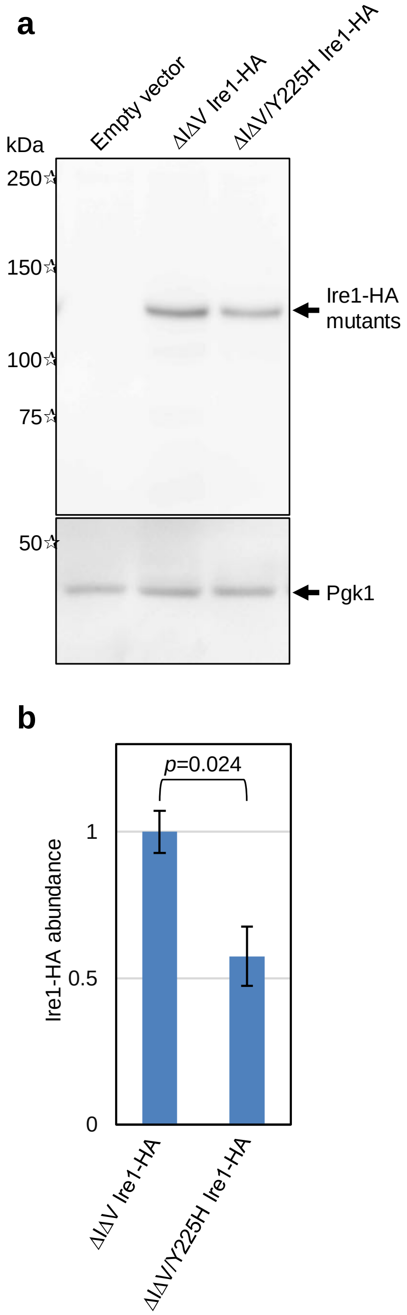

Figure S2 Effect of the Y225H mutation on cellular abundance of ΔIΔV Ire1
(a) KMY1015 cells (*ire1Δ*) transformed with the Ire1-HA-expression plasmid pRS315-IRE1-HA carrying the indicated mutations (or the empty vector pRS315) were grown at 30°C in SD medium, and lysates of cells (equivalent to OD_{600} = 0.25) were analyzed by SDS-PAGE (10% acrylamide) and anti-HA Western blotting. The anti-Pgk1 immunoblot serves as a loading control. (b) The experiment shown in the panel (a) was performed with multiple biological samples. Band densities were quantified, are normalized against that of ΔIΔV Ire1-HA cells, and are presented.

Figure S3

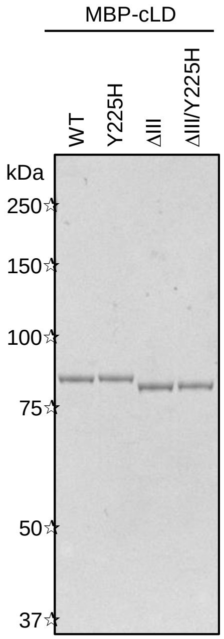

Figure S3 MBP-cLD and its mutants used in Fig. 3.
Bacterially expressed MBP-cLD (wild-type: WT) or its mutants (0.25 μg each) were purified, were run on 8% SDS-PAGE gel and were stained with Coomassie blue.

Figure S4

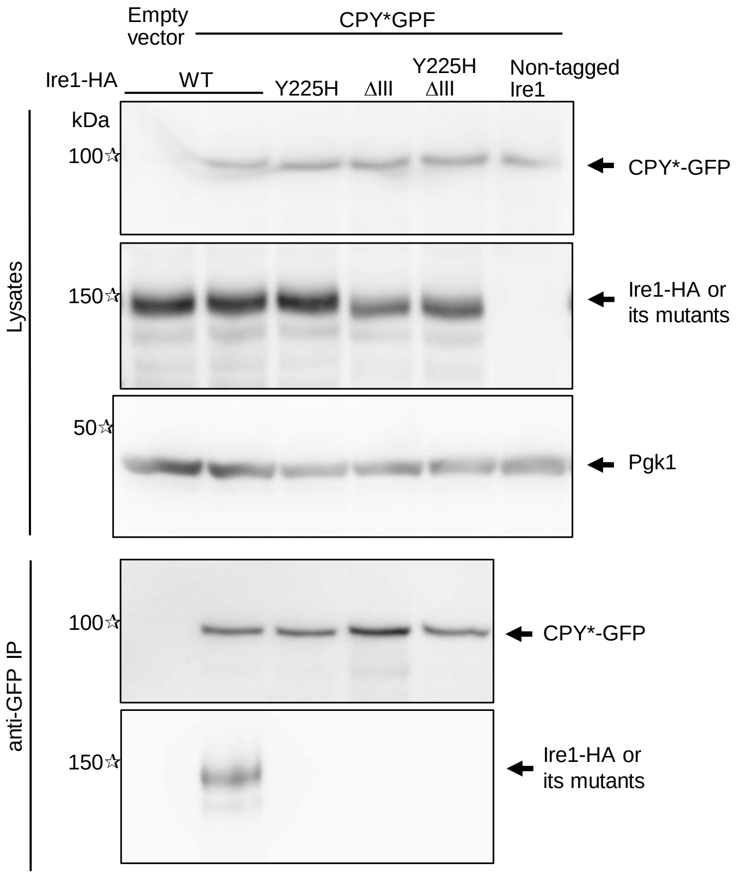

Figure S4 *In vivo* interaction between Ire1 and a model ER-located unfolded protein is abolished by the ΔIII or the Y225H mutation

KMY1015 cells (*ire1Δ*) carrying the *GAL1* promoter-driven CPY*-GFP expression plasmid pRS313-GAL1pr-CPY*-GFP (or the empty vector pRS313) and the Ire1-HA-expression plasmid pRS426-IRE1-HA (or its mutant versions) were cultured in the presence of 2% galactose to induce the CPY*-GFP expression. After harvest, cells were treated with the protein crosslinker DSP, were lysed, and were subjected to anti-GFP immunoprecipitation. Cell lysates (equivalent to OD_{600}=0.25 cells) and anti-GFP immunoprecipitants (anti-GFP IP; equivalent to OD_{600}=10 cells) were then analyzed by Western blotting using the anti-GFP, anti-HA or anti-Pgk1 antibody. The blot images are cropped, and the uncropped blots are presented in supplementary Figs S13 and S14.

Figure S5

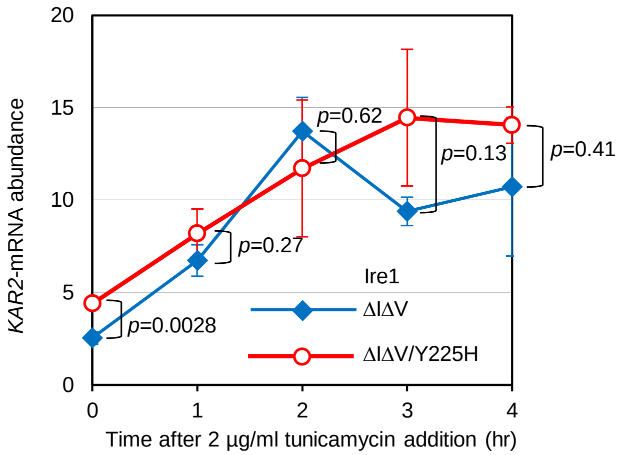

Figure S5 Induction of the *KAR2* gene upon tunicamycin exposure in cells carrying ΔIΔIIIΔV or ΔIΔIIIΔV/Y225H Ire1

After being grown at 30°C in SD medium, KMY1015 cells (*ire1Δ*) carrying the indicated mutant versions of *IRE1* plasmid pRS313-IRE1 were ER-stressed by tunicamycin (2 µg/ml), and were checked for the *KAR2*-mRNA abundance using the RT-qPCR technique. The resulting values are normalized against that of unstressed KMY1015 cells carrying wild-type pRS313-IRE1, which is set at 1.0.

Figure S6

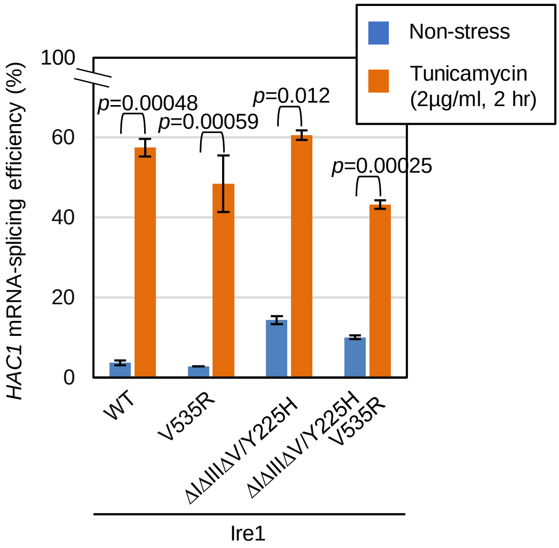

Figure S6 Effect of the V535R mutation on the *HAC1* mRNA-splicing efficiency
After being grown at 30°C in SD medium, KMY1015 cells (*ire1Δ*) carrying the *IRE1* plasmid pRS313-IRE1 (wild-type: WT) or its mutants were ER-stressed by tunicamycin (2 µg/ml, 2 hr) or remained unstressed, and were checked for the *HAC1* mRNA-splicing efficiency using the RT-PCR technique.

Figure S7

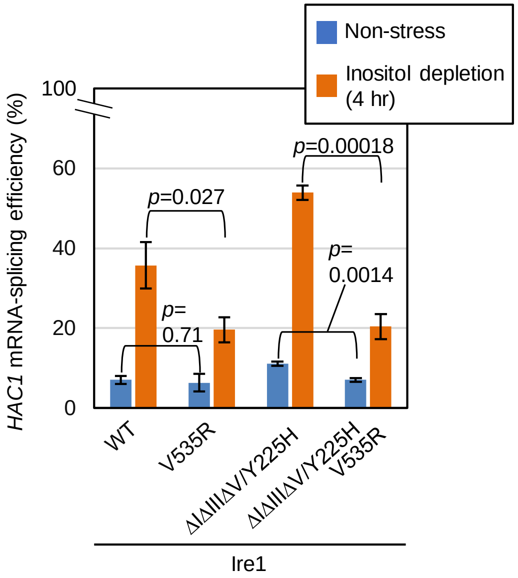

Figure S7 Induction of the *HAC1*-mRNA splicing upon inositol depletion in cells carrying wild-type or ΔIΔIIIΔV/Y225H Ire1

After being grown at 30°C in SD medium, KMY1015 cells (*ire1Δ*) carrying the *IRE1* plasmid pRS313-IRE1 (wild-type: WT) or its mutants were shifted to inositol depletion medium and further cultured for 5 hr, or remained unstressed. Then they were checked for the *HAC1* mRNA-splicing efficiency using the RT-PCR technique.

Figure S8

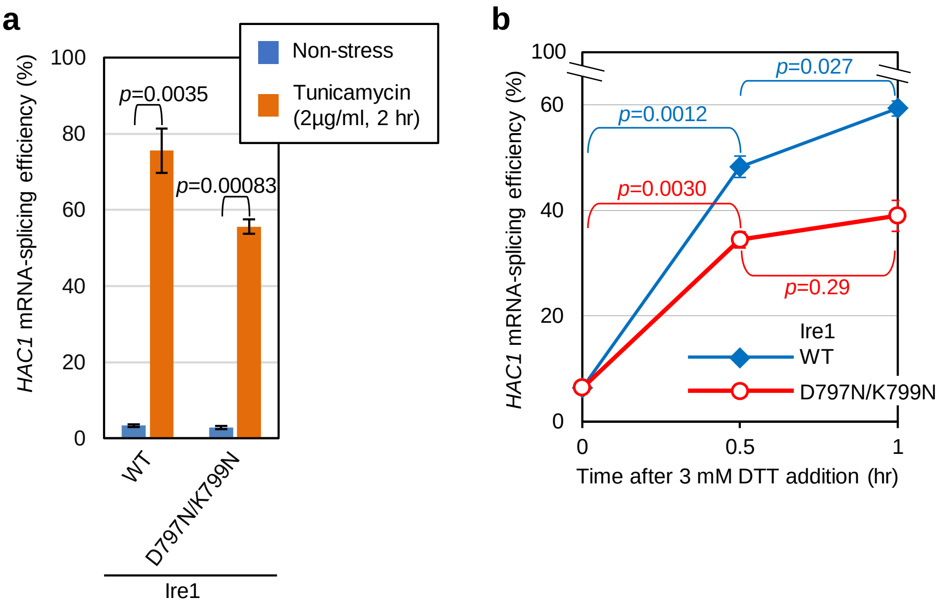

Figure S8 Responsiveness of D797N/K799N Ire1 to potent ER-stress stimuli

After being grown at 30°C in SD medium, KMY1015 cells (*ire1Δ*) carrying the *IRE1* plasmid pRS313-IRE1 (wild-type: WT) or its D797N/K799N mutant were ER-stressed as indicated or remained unstressed, and were checked for *HAC1* mRNA-splicing efficiency using the RT-PCR technique.

Figure S9

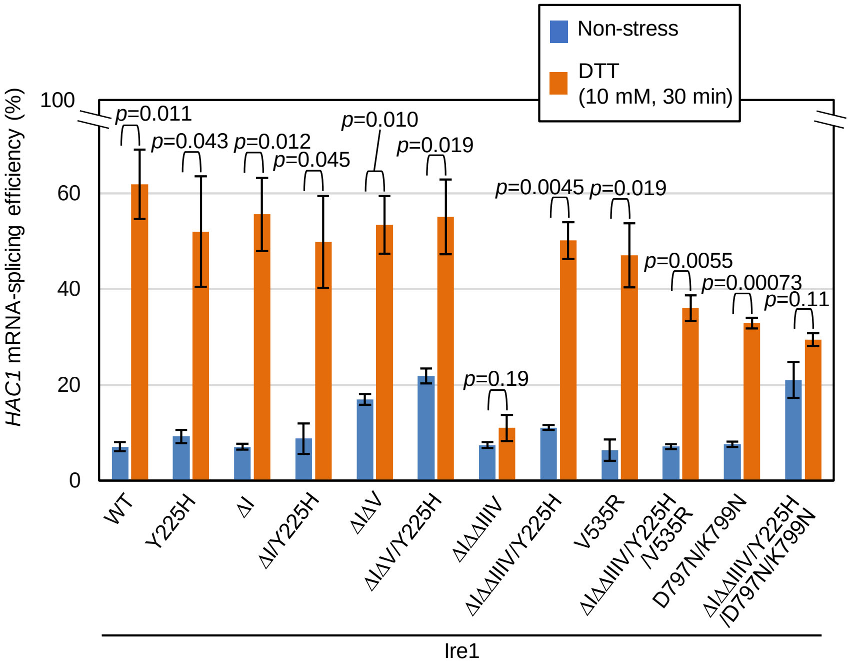

Figure S9 Responsiveness of Ire1 mutants to 10 mM DTT

After being grown at 30°C in SD medium, KMY1015 cells (*ire1Δ*) carrying the *IRE1* plasmid pRS313-IRE1 (wild-type: WT) or its mutants were ER-stressed by DTT (10 mM, 30 min) or remained unstressed, and were checked for the *HAC1* mRNA-splicing efficiency using the RT-PCR technique.

Figure S10

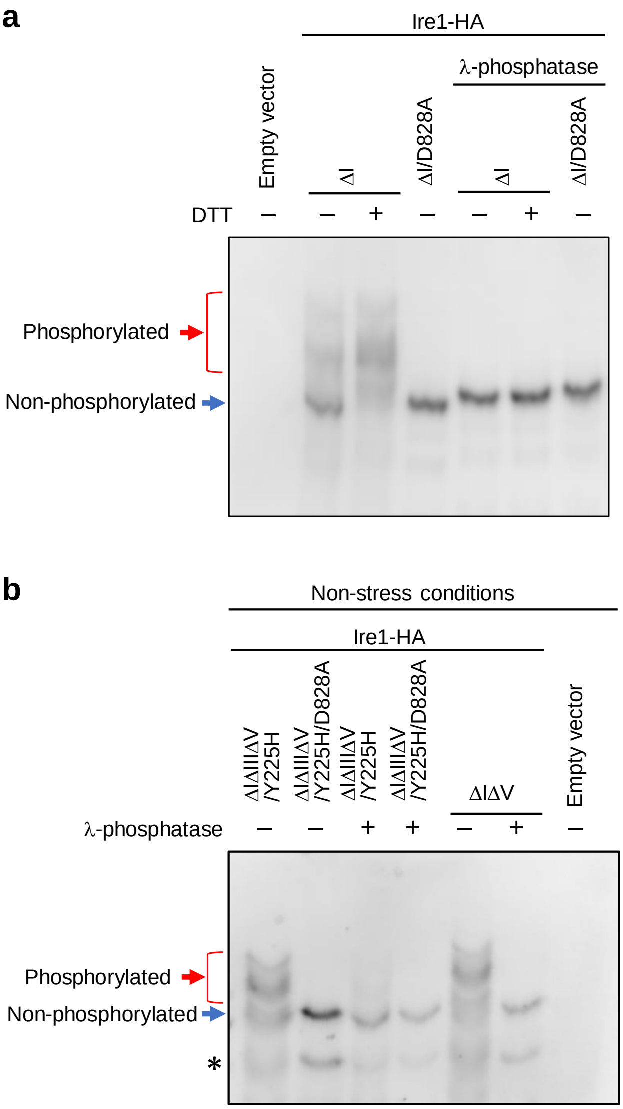

Figure S10 λ-protein phosphatase treatment of the Phos-tag SDS-PAGE samples
After being grown at 30°C in SD medium, KMY1015 cells (*ire1Δ*) carrying the indicated mutant versions of the Ire1-HA expression plasmid pRS315-IRE1-HA (or the empty vector) were cultured in the presence of DTT (10 mM, 30 min) or remained unstressed. Lysates of cells (equivalent to OD_{600} = 0.25) were treated (or not treated) with λ-protein phosphatase as done in Ref. 30, and were analyzed by Phos-tag SDS-PAGE and anti-HA Western blotting. *: partial degradation product of the Ire1-HA mutants.

Figure S11

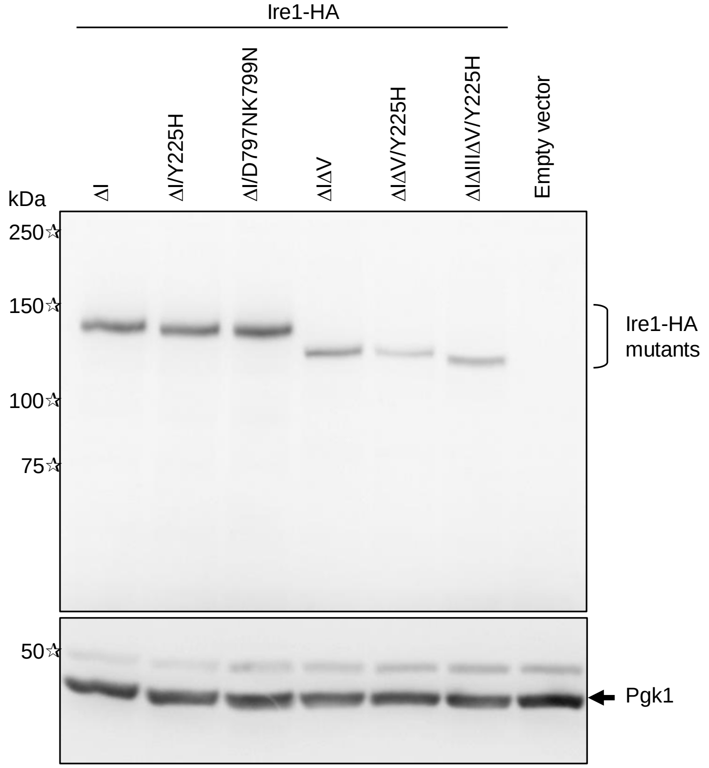

Figure S11 Detection of the Ire1-HA mutants through normal SDS-PAGE and anti-HA Western blotting

KMY1015 cells (*ire1Δ*) transformed with the Ire1-HA-expression plasmid pRS315-IRE1-HA carrying the indicated mutations (or the empty vector pRS315) were grown at 30°C in SD medium, and lysates of cells (equivalent to OD_{600} = 0.25) were analyzed by SDS-PAGE (10% acrylamide) and anti-HA Western blotting. The anti-Pgk1 immunoblot serves as a loading control.

Figure S12

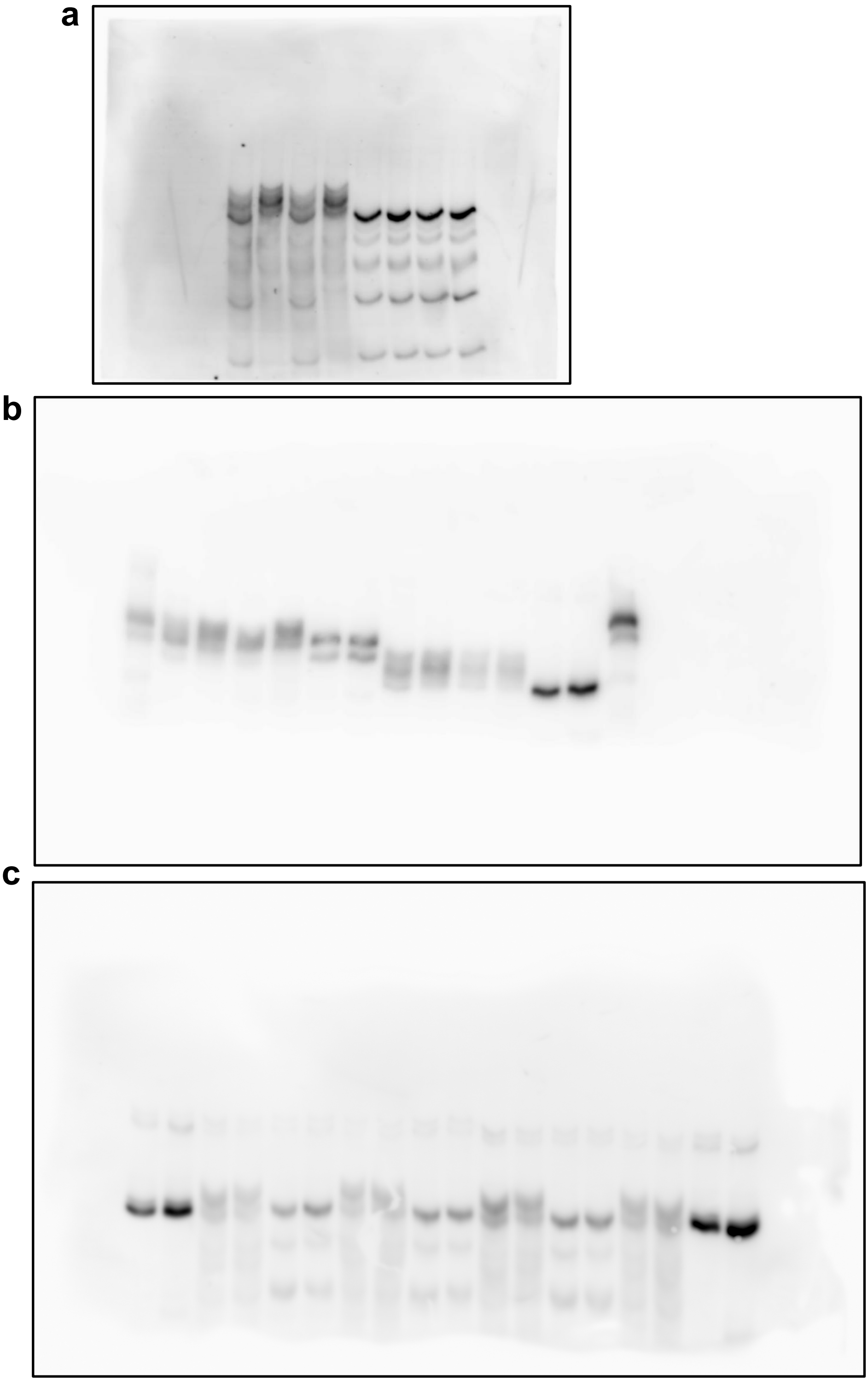

Figure S12 Uncropped immunoblot images shown in Fig. 5
They are original images that have not been electrically modified by any image-processing software.

Figure S13

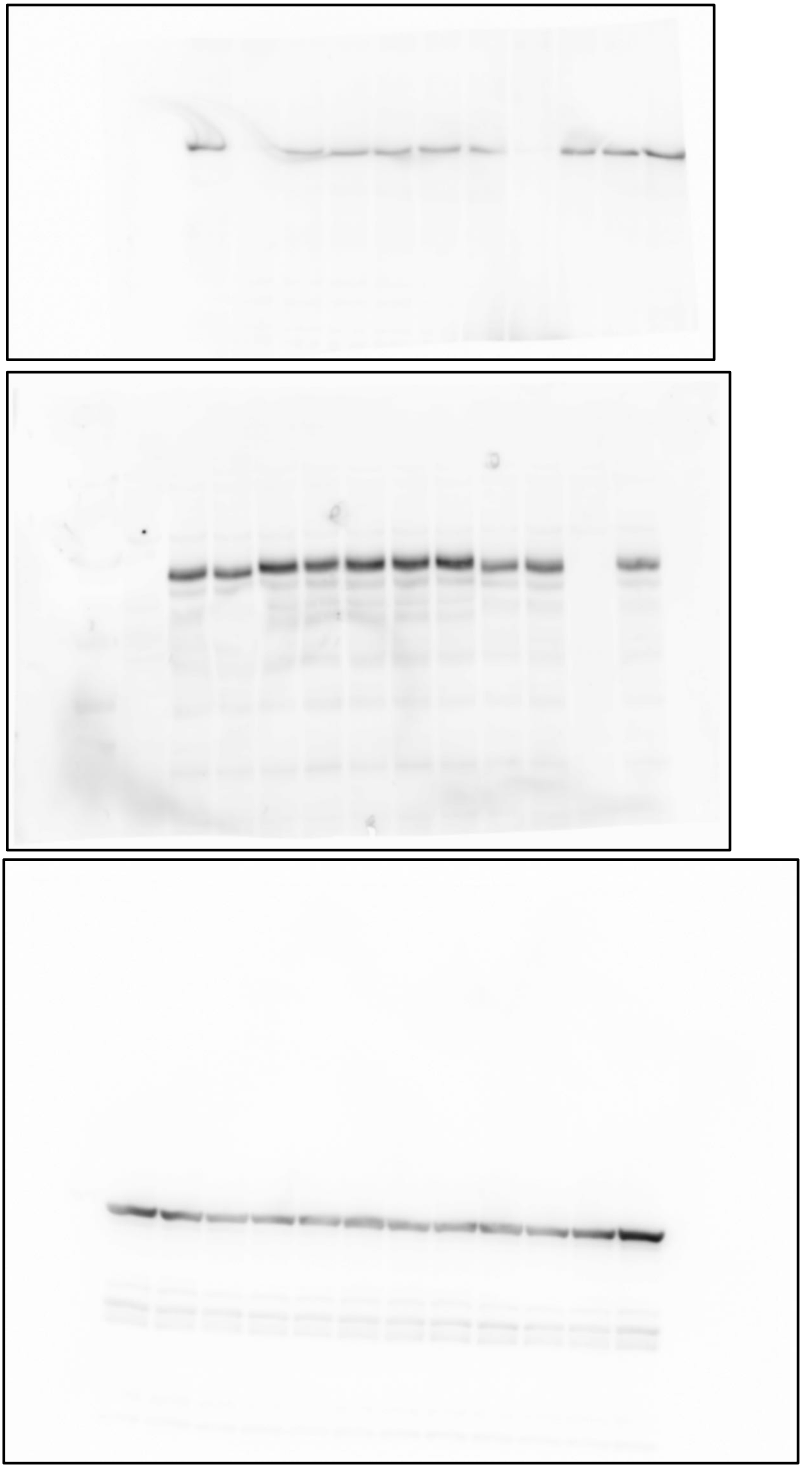

Figure S13 Uncropped immunoblot images shown in Fig. S4
They are original images that have not been electrically modified by any image-processing software.

Figure S14

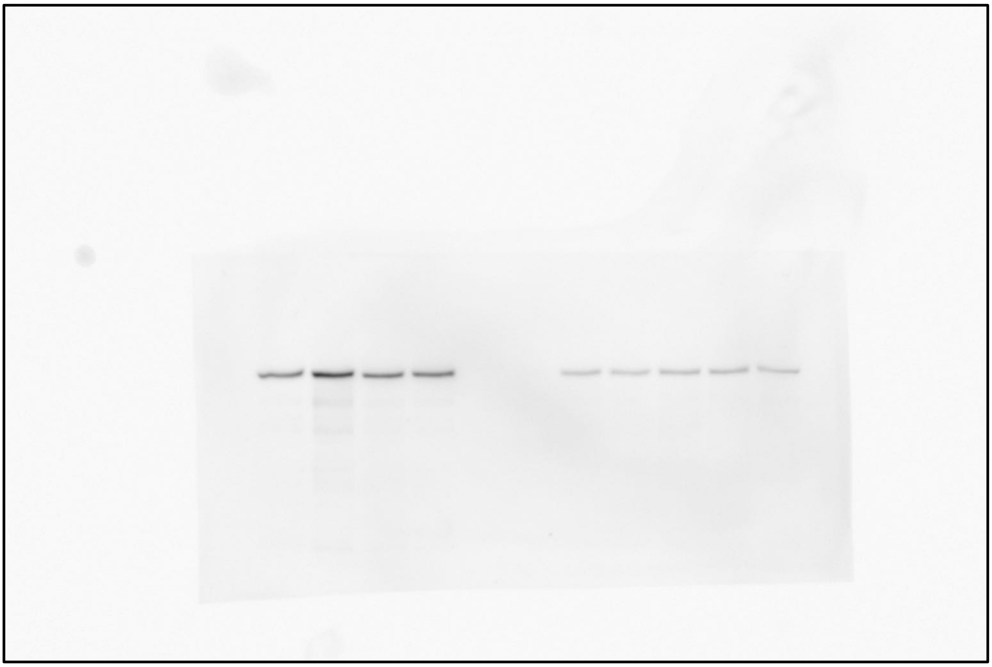

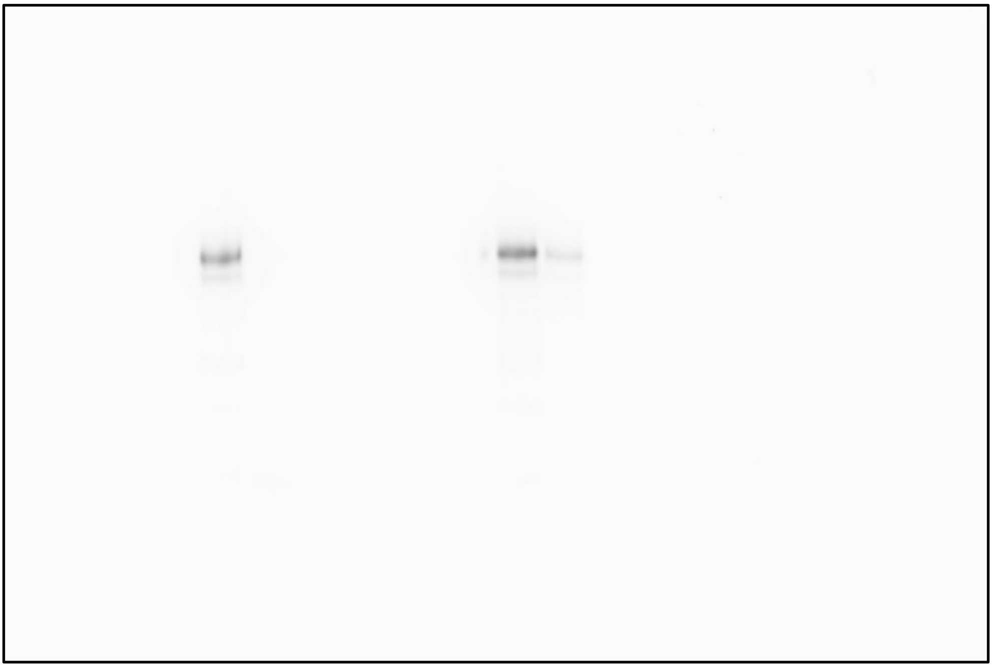

Figure S14 Uncropped immunoblot images shown in Fig. S4
They are original images that have not been electrically modified by any image-processing software.
